# Measuring neurodevelopment of inhibitory control in children using naturalistic virtual reality

**DOI:** 10.1038/s41598-025-10974-3

**Published:** 2025-07-24

**Authors:** Larisa-Maria Dina, Paola Pinti, Tim J. Smith

**Affiliations:** 1 Centre for Brain and Cognitive Development, Birkbeck, University of London, London, WC1E 7JL UK; 2 Department of Psychology, King’s College London, London, SE5 8AB UK; 3 Department of Medical Physics and Biomedical Engineering, University College London, London, UK; 4 Creative Computing Institute, University of the Arts London, London, WC1V 7EY UK

**Keywords:** Naturalistic, Virtual reality, CAVE, fNIRS, Toddlers, Development, Inhibitory control, Neuroscience, Cognitive neuroscience, Development of the nervous system

## Abstract

Inhibitory control develops over time and is linked to fronto-striatal maturation. Traditional computerised assessments often lack ecological validity and are not age appropriate. Here, we developed a naturalistic, age-appropriate paradigm using a cave automatic virtual environment (CAVE) and mobile functional near-infrared spectroscopy (fNIRS) to measure response inhibition in children aged 3–7-years. The new task was validated in adults (N = 24, M_age_ = 30.38, SD = 10.54), and children (N = 36, M_age_ = 4.44, SD_age_ = 1.11 years). Participants completed two Go/No-Go tasks: a standard computer-based version and an adapted CAVE version, while fNIRS recorded brain activity in the bilateral dorsolateral frontal cortices. The aims were to compare behavioural performance in the CAVE and computer tasks, establish if the tasks capture developmental differences in inhibitory control, assess their psychometric properties (convergent and divergent validity), determine the feasibility and acceptability of the multimodal CAVE-fNIRS setup in early childhood, and characterise the neural correlates of response inhibition in both 2D and 3D tasks. Consistent with typical Go/No-Go tasks, we found higher error rates in mixed blocks compared to Go-only blocks. No significant correlations were found between self-reported (adults) or parent-reported (children) inhibition measures and task performance, nor between performance metrics across the CAVE and computer tasks, though children generally exhibited poorer performance across most metrics compared to adults, reflecting the prolonged developmental trajectory of inhibitory control. The novel CAVE task proved feasible and acceptable, with high completion rates and absent or minimal virtual reality-induced symptoms. Specific to the early childhood sample, Go/No-Go blocks in the CAVE task elicited higher activity in the left inferior frontal gyrus. This study shows the brain correlates of response inhibition during unrestricted movement in 2D and 3D settings in young children, integrating age-appropriate fNIRS with an immersive CAVE, opening potential new approaches to studying neurodevelopment.

**Supplementary Information:**

The online version contains supplementary material available at 10.1038/s41598-025-10974-3.

## Introduction

Inhibitory control is a core executive function and is often used as an umbrella term to refer to the multiple facets of inhibition, including response inhibition^1^. Response inhibition refers to the ability to actively suppress or delay prepotent responses and replace them with context-appropriate responses to fulfil a goal^2^. Inhibitory control follows a long developmental trajectory as it emerges near the end of the first year of life, rapidly matures throughout early childhood, and continues to develop throughout adolescence until early adulthood^3,4^. This prolonged developmental trajectory is closely linked to the maturation of brain structures and neural networks^5^. Specifically, meta-analytic evidence highlights the existence of an inhibitory processing network system where response inhibition activates a fronto-striatal system^6^. The association between response inhibition and the structural and functional development of the prefrontal cortex (PFC) is further evidenced by multimodal neuroimaging studies using inhibition tasks^7^.

Consistent with its role in goal-directed behaviour in everyday activities, there are widespread standardised assessments of response inhibition^7^, some of which have also been adapted to be used in neuroimaging studies^7^ and across the lifespan^8^. There are multiple ways in which these assessments can be delivered, ranging from pen and paper questionnaires^9^ to tasks or questionnaires delivered on computers, tablets or smartphones^10–14^. Nonetheless, despite multiple assessment delivery modalities and its importance in everyday behaviours, response inhibition is typically assessed using non-naturalistic assessments. Non-naturalistic assessments can be conceptualised on the latter end of a continuum using decontextualised, static, repetitive stimuli, largely delivered in constrained laboratory settings^15,16^. While it is important that such tasks are domain-specific and able to measure their target construct (convergent validity), filtering out noise or confounds in favour of isolating latent variables means that the complexities of everyday life are not adequately captured^17^. This is important because real-life situations where the suppression or delay of prepotent responses is expected involve both task relevant and task irrelevant information, depending on the context we are in, and the goal we aim to achieve. One of the directions proposed to move the field towards more naturalistic research is bringing more realistic stimuli into the laboratory^16,18^. This allows for the isolation of latent variables while introducting curated noise, which is possible through immersive virtual reality (VR). Tasks that use VR are characterised by interactive and immersive elements produced using advanced computer technologies to create realistic environments in 3D^19^. Inhibitory control tasks delivered through VR have been shown to have comparable psychometric properties with equivalent computerised versions, and be acceptable and feasible for participants, with generally few reported cybersickeness symptoms and high completion rates^16^. Increasing the ecological validity of cognitive tasks is crucial, as performance on neuropsychological tests has been shown to account for only 4.6–21.4% variance in daily functioning^20^. Furthermore, an intrinsic property of naturalistic methods is that they are intuitive and contribute to a level of enjoyment, therefore increasing participant engagement^21^ and making them attractive for populations that are naturally more difficult to engage in research (e.g., children, clinical populations^22^).

Despite that virtual reality-based cognitive tasks have started to become increasingly used in neuropsychological assessment^19^, there are several angles that remain largely unexplored. Firstly, research using VR methods focusing on children continues to be relatively scarce^16,23^. This is a missed opportunity because children naturally engage in play, and immersive VR can provide children with multisensory experiences which may either replicate scenes from the physical world or create fictious scenarios, much like the settings where play occurs or that are imagined during play. Secondly, most research using neuropsychological tests in VR in developmental samples has evaluated the Virtual Classroom^24^, a continuous performance test embedded in a virtual setting that most young people are familiar with—a school classroom. The Virtual Classroom system has been shown to have high ecological validity, as it simulates a real-world classroom, and there is evidence to suggest that it can discriminate between children with Attention Deficit Hyperactivity Disorder (ADHD) from matched controls^25,26^. While it is important that attention and cognitive control are measured in a real-world context where school children spend a significant portion of their day, VR has rarely been used to assess inhibitory control in children under the age of 7, for whom play contexts might be more salient^27^. To our knowledge, only two studies to date have used immersive VR in early childhood. One study compared the effect of different technologies such as immersive VR and television on inhibitory control skills, social compliance and sharing in four- to six-year-olds^28^. They used a Simon Says task in both conditions and found that children were less likely to suppress their motoric response in the VR condition, indicating that VR might elicit differential cognitive responses compared to less immersive technologies. The second study developed and validated an immersive VR platform to assess social development in three- to five-year-olds whilst simultaneously recording brain activity using a mobile functional near-infrared spectroscopy (fNIRS) system, showing the feasibility of using this setup to assess social preferences in naturalistic settings^29^. Nonetheless, none of the studies to date used an immersive cave automatic virtual environment (CAVE) to study inhibitory control. Finally, no study to date has assessed the neural correlates of inhibitory control in virtual environments. Existing paediatric studies assessing the neural correlates of response inhibition using fNIRS during standard tasks report recruitment of the right dorsolateral prefrontal cortex in children aged 4–10 years^5^ and children aged 7–12 years^30^, stronger connections between left frontal and parietal cortices^31^, recruitment of the right middle and inferior frontal gyri and bilateral supramarginal gyri in children aged 4–7 years^32^, and increased functional activation in the right prefrontal and parietal cortices as early as at 10 months of age^33^. In addition to the frequently reported involvement of the right prefrontal areas in inhibitory control, the left inferior frontal gyrus (IFG) is also critical for response inhibition, as evidenced in functional magnetic resonance imaging (fMRI) studies in adults, e.g., in patients with focal damage in the left IFG^34^ and in charactising areas of absolute diversity for executive functions in healthy adults^35^.

Despite a rapid maturation of inhibitory control in early childhood, it remains challenging to reliably measure the neural correlates of response inhibition in paediatric populations. Most existing imaging techniques are not appropriate for young children due to noise and movement restrictions (e.g., fMRI), and those that are age-appropriate (e.g., electroencephalography) have limited spatial resolution, making it difficult to identify neuroanatomical regions associated with response inhibition^36^. More broadly, there is a lack of age-appropriate assessments for assessing this construct, and many of the traditional executive function measures are designed for adult populations and adapted for children^37,38^. Furthermore, to date it remains unclear if response inhibition may have different neural and behavioural characteristics during more naturalistic behaviour, such as during a VR inhibitory control task. To our knowledge, no study to date used a multimodal fNIRS-VR setup during a more naturalistic inhibitory control task, and only one study compared a VR task with a 2D equivalent in young children^28^. In that study, authors reported that children were more disinhibited in the VR condition of their Simon Says inhibitory control task, possibly influenced by the salience of the VR environment, but the neural correlates of this behaviour were not measured.

To address the age-appropriateness and ecological validity challenges highlighted above, the aim of the current study is to develop a novel ecologically valid paradigm for the assessment of response inhibition in a CAVE environment, a cubic room where virtual scenes are projected on the three walls surrounding the participants and on the floor. The CAVE is well-suited for children for several reasons. First, the tracked stereoscopic glasses worn in a CAVE are currently reported to weigh five to ten times less than a typical head-mounted display (HMD), and they can be custom 3D-printed to create child-appropriate sizes^39^. This is an important consideration when conducting research with young children. Second, a fundamental difference between CAVE and HMDs is the eye-to-screen distance, and the fixed distance of HMDs has been reported to impact distance perception^40^. To this point, CAVEs allow users to see their own bodies and other physical cues in their environment (e.g., screen edges), to estimate distances in the virtual world^41^, make them feel safer and protect against safeguarding concerns^42^. Finally, this virtual environment is appropriate for recreating naturalistic settings from real life into a controlled laboratory environment, therefore allowing us to introduce task-irrelevant information (i.e., contextual information, distractors) to the paradigm whilst also customising the context and stimuli for the population being tested.

To further understand the neural correlates underlying response inhibition in naturalistic settings, we combined the novel CAVE system with mobile fNIRS. fNIRS is a non-invasive neuroimaging technique utilising near-infrared light to measure concentration changes in oxygenated (HbO_2_) and deoxygenated (HbR) haemoglobin. This indirect measurement of functional brain activity is achieved by placing a certain number of light sources on the scalp, capable of penetrating into the brain tissue, as well as optical detectors to collect back-scattered light^43^. It is based on the principle of neurovascular coupling, referring to the oversupply of cerebral blood flow to active brain regions to meet the increase in oxygen demand following neuronal firing^44^. It is suggested that concentration changes in oxygenated and deoxygenated blood are closely related to neuronal activity in the cerebral tissue^44^. Importantly for paediatric samples, fNIRS systems are lightweight, can be wireless and can tolerate a large degree of motion, therefore allowing for less unrestrained movement. These characteristics make fNIRS suitable for young children who are not able to remain still for long periods of time^45^ and are also conducive to more naturalistic testing in cognitive science^46^. Based on the neural correates of response inhibition previously reported in children and adults in standardised tasks, we decided to record brain activity in the bilateral frontal cortices.

The aims of this proof-of-principle study were multifold. Our first aim was to compare behavioural performance in a computerised Go/No-Go task with that in a naturalistic Go/No-Go task in a CAVE in two separate samples of adults and young children – this was firstly done in adults to establish the feasibility of the multimodal fNIRS-CAVE setup, and then in children aged 3–7 years. To this aim, we hypothesised there will be higher error rates in Mixed blocks compared with Go blocks in both adults and children, as in typical for Go/No-Go tasks^47,48^, and that these differences will be more pronounced in the novel CAVE task. To our knowledge, this is the first study assessing inhibitory control in a CAVE setting against a 2D comparator, with only one previous study measuring the same construct using an inhibition task delivered through either VR headsets, or a 2D comparator (a TV). Therefore, the expectation that behavioural performance would be poorer on the CAVE task is informed by the results reported by Bailey et al.^28^, who found that children exhibited lower inhibition in the VR condition. Further related to performance metrics, we also expected to see longer reaction times in Go blocks in the novel CAVE task, since the task is more visually and motorically complex. Our second aim was to establish if the novel CAVE task is suitable for capturing developmental differences in inhibitory control. To this aim, we hypothesised that age is positively associated with better performance on the CAVE task, in line with the long developmental trajectory of inhibitory control^4,5^. Our third aim was to assess the psychometric properties of the task (convergent and divergent validity) and determine the feasibility and acceptability of the CAVE task in conjunction with fNIRS in early childhood. To determine convergent validity, we hypothesised there would be associations between behavioural performance in the CAVE and computer task and self- or parent-reports on constructs relevant for inhibitory control (e.g., externalising symptoms, associated with lower inhibition^49^). To assess divergent validity, we did not expect to find correlations between behavioural performance and self- or parent-reports of other executive functioning domains (e.g., working memory, cognitive flexibility). To assess the feasibility of a multimodal CAVE-fNIRS setup, we reported study completion rates and to assess the acceptability of the novel CAVE task, we measured possible VR-induced symptoms and effects following the task. Lastly, we aimed to characterise the neural correlates of response inhibition in during a 2D and immersive 3D task, and assess associations between neural activity and behavioural performance. Here, we predicted that functional brain activity would be localised in areas commonly associated with inhibitory control, i.e., in the frontal regions, and that there would be stronger activation patterns during Mixed blocks compared with Go blocks.

## Results

36 children (36.1% female, M_age_ = 4.44, SD_age_ = 1.11 years) and 24 adults (58.3% female, M_age_ = 30.38, SD = 10.54) were included in the analyses of the behavioural data.

### Behavioural analyses

#### Task performance in adults (aim 1)

##### Within tasks

**Fig. 1:**
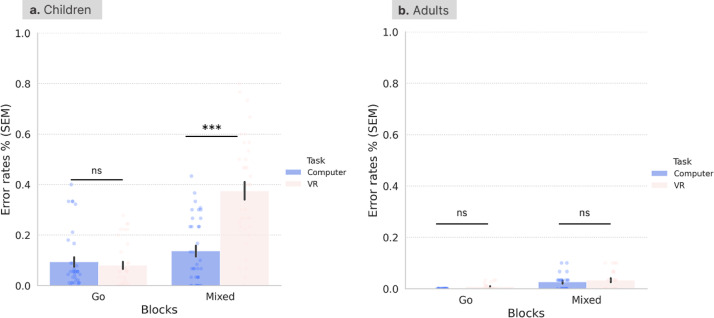
Error rates (%) in Go and Mixed blocks for the standardised computer-based task and the novel CAVE task in (**a**) toddlers and pre-schoolers, and (**b**) adults. Error bars represent the standard error of the mean (SEM). Wilcoxon rank tests were used to compare error rates between the two tasks for each group. ***, *p* < 0.001; ns, not significant.

To address the first aim, we first investigated task performance separately in the CAVE and computer task in the adult sample. Due to the non-parametric nature of this data, we used the Wilcoxon rank test and a bootstrapping procedure to calculate the 95% confidence intervals. Error rates were significantly higher in mixed blocks (M = 0.03, SD = 0.04) compared with Go blocks (M = 0.01, SD = 0.01) in the CAVE task (Z = − 2.81 *p* = 0.01) [95% CI(0.002, 0.004)], and in mixed blocks (M = 0.03, SD = 0.03) compared with Go blocks (M = 0.00, SD = 0.00) in the standardised computer task (Z = − 3.12, *p* = 0.002) [95% CI (0.000, 0.001)] (Fig. 1).

##### Across tasks

**Fig. 2:**
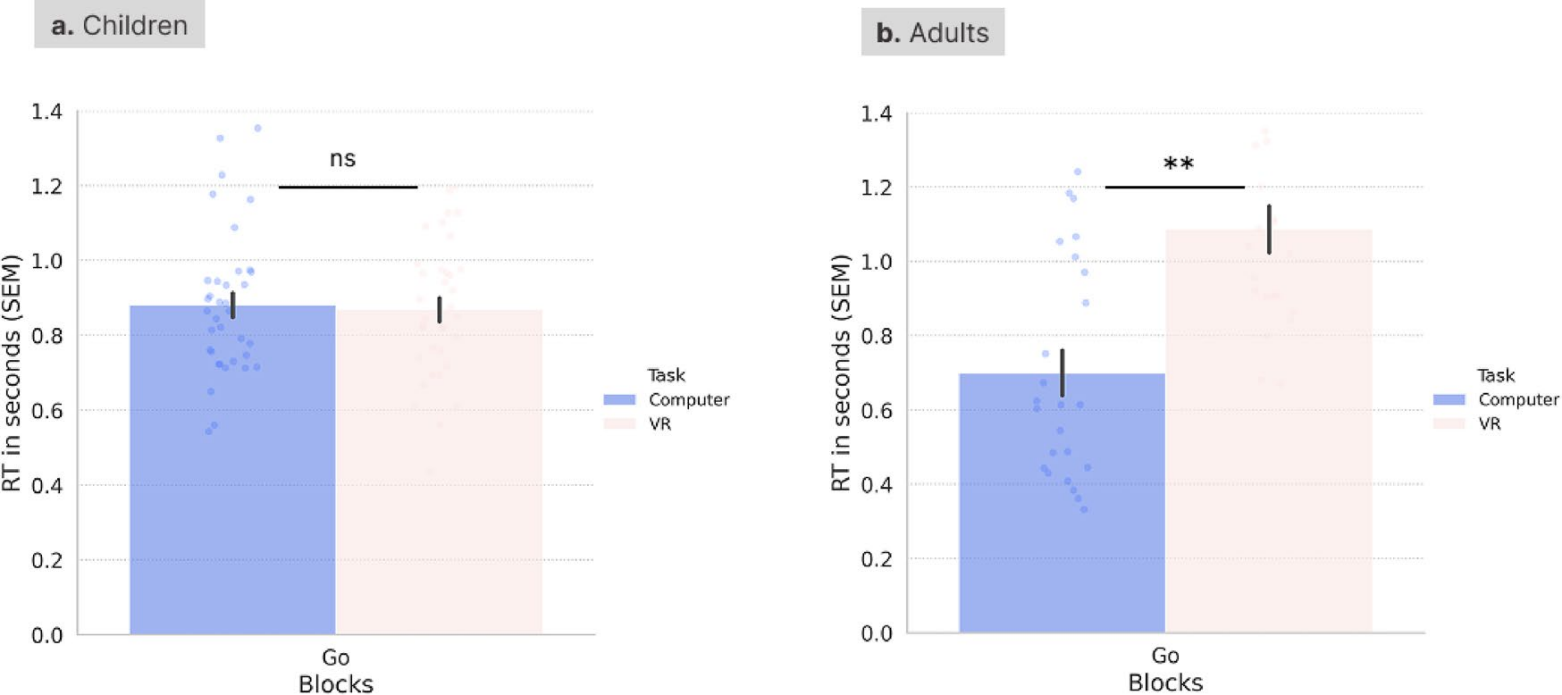
Reaction times (seconds) in Go blocks for the standardised computer-based and the novel CAVE task in (**a**) toddlers and pre-schoolers, and (**b**) adults. Error bars represent the standard error of the mean (SEM). Wilcoxon rank tests were used to compare RT between the two tasks for each group. **, *p* < 0.01; ns, not significant.

Task performance metrics were also compared across the two tasks in adults using Wilxocon rank tests for paired non-parametric data, and bootstrapping to calculate 95% confidence intervals. Error rates were significantly different in the Go blocks (Z = − 2.54, *p* = 0.01) [95% CI(0.006, 0.010)], with slightly higher error rates in the CAVE task (M = 0.008, SD = 0.01) than in the computerised task (M = 0.000, SD = 0.00). There were no differences in Mixed blocks (Z = − 0.79, *p* = 0.43) [95% CI(0.457, 477)] (Fig. 1) between the computerised (M = 0.026, SD = 0.03) and CAVE (M = 0.033, SD = 0.04) tasks. Comparing reaction times in Go blocks between the two tasks, we found higher reaction times in the novel CAVE task (M = 1.09, SD = 0.29) compared with the computerised task (M = 0.70, SD = 0.30), Z = − 3.23, *p* < 0.001 [95% CI(0.000, 0.001)] (Fig. 2).

#### Task performance in children (aim 1)

##### Within tasks

To address the first aim, in a second step we investigated task performance separately in the CAVE task and, respectively in the computer task in the children sample, using the Wilcoxon rank test for related, non-parametric data and a bootstrapping procedure to calculate 95% confidence intervals. Error rates were significantly higher in mixed blocks (M = 0.38, SD = 0.21) compared with Go blocks (M = 0.08, SD = 0.09) in the CAVE task (Z = − 5.19, *p* < 0.001) [95% CI(0.000,000)]. Nontheless, in contrast to the results from the adult sample, in children the difference between error rates in mixed blocks (M = 0.14, SD = 0.13) and Go blocks (M = 0.09, SD = 0.11) in the standardised computer task was not significant (Z = − 1.89, *p* = 0.07) [95% CI(0.062, 0.72)] (Fig. 1).

##### Across tasks

Task performance metrics were also compared across the CAVE and computer task in children. In children, error rates in the Mixed blocks were significantly different in the CAVE (M = 0.38, SD = 0.21) and computerised task (M = 0.14, SD = 0.13) (Z = − 4.74, *p* < 0.001) [95% CI(0.000, 0.000)]. There were no differences in Go blocks (Z = − 0.12, *p* = 0.91) [95% CI(0.907,0.918) between the computerised (M = 0.09, SD = 0.11) and CAVE (M = 0.08, SD = 0.09) tasks. Comparing reaction times in Go blocks between the two tasks in the early childhood sample, we did not find a significant difference between the reaction times in the novel CAVE task (M = 0.87, SD = 0.18) compared with the computerised task (M = 0.88, SD = 0.19), Z = − 0.51, *p* = 0.61 [95% CI(0.619,0.638)] (Fig. 2).

#### Developmental differences in task performance (aim 2)

Since both tasks were designed to be age-appropriate for young children, we investigated if they captured neurodevelopmental differences. To this end, Mann–Whitney U tests were computed using group (adults or children) as the between-subject variable, and task performance in each task as the dependent variable. Error rates in the novel CAVE task were significantly higher in the children group, as expected, for Go (Children: M = 0.08, SD = 0.09; Adults: M = 0.01, SD = 0.01, Z = − 4.29, *p* < 0.001, 95% CI[0.000,000]) and mixed blocks (Children: M = 0.38, SD = 0.21; Adults: M = 0.03, SD = 0.04, Z = − 5.94, *p* < 0.001, 95% CI[0.000,0.000]), and in the standard computerised task for Go (Children: M = 0.09, SD = 0.11; Adults: M = 0.00, SD = 0.00, Z = − 6.34, *p* < 0.001, 95% CI[0.000,0.000]) and mixed blocks (Children: M = 0.14, SD = 0.13; Adults: M = 0.03, SD = 0.03, Z = − 3.45, *p* < 0.001, 95% CI[0.000,0.001]). Interestingly, adults were significantly quicker in Go blocks in the computer task (Adults: M = 0.72, SD = 0.30; Children: M = 0.87, SD = 0.19, Z = − 2.61, *p* = 0.009, 95% CI[0.006,0.010]) but slower in Go blocks in novel CAVE task (Adults: M = 1.09, SD = 0.29; Children: M = 0.87, SD = 0.18, Z = − 2.71, *p* = 0.007, 95% CI[0.004,0.007]) (Figs. 1 and 2).

##### Age effects (aim 2)

**Fig. 3:**
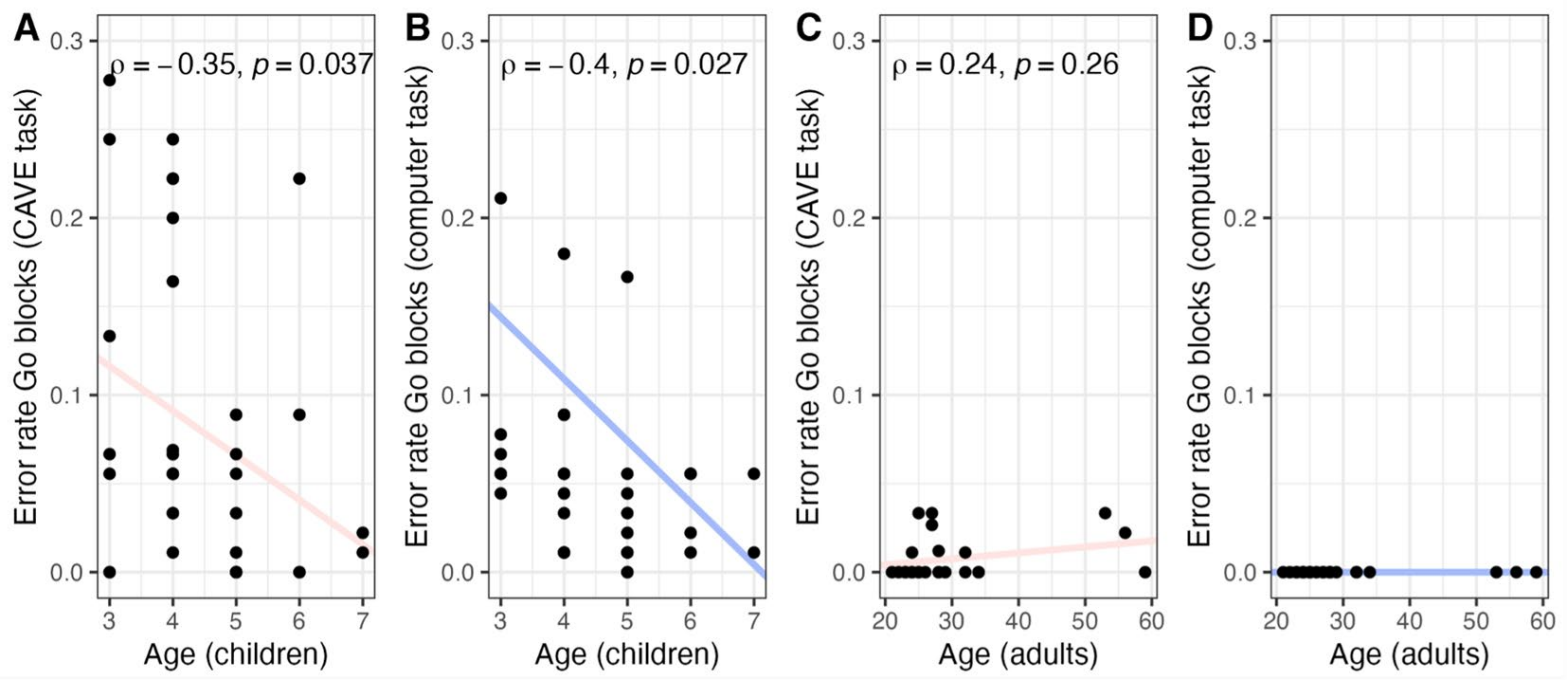
Error rates in Go blocks and participants’ age. Spearman’s correlation coefficients are denoted by the letter ρ (rho), and statistically significant associations are these where *p* < 0.05 (uncorrected). FDR-corrected *p*-values are presented in the text and used for interpretation.

To further investigate the validity of the novel inhibitory control CAVE task, we correlated error rates in Go blocks, a measure of task performance, with participants’ age in years for each group (Fig. 3). In children, error rates showed negative associations with age in both tasks, such that being older was linked to better performance, though these correlations did not survive FDR correction for multiple comparisons (CAVE task: rho = − 0.35, p_uncorrected_ = 0.037/p_FDR corrected_ = 0.07; CB task: rho = − 0.40, p_uncorrected_ = 0.027/p_FDR corrected_ = 0.07) (Fig. 3A,B). In adults, error rates in Go blocks were not significantly correlated with age in either task, as expected due to the tasks being designed for children and therefore easy for this age category (CAVE task: rho = 0.24, p_uncorrected_ = 0.26/p_FDR corrected_ = 0.25) (Fig. 3C). Error rates in Go blocks in the computer task were 0 for all participants, such that no statistics were computed (Fig. 3D).

### Psychometric properties and user experience

#### Convergent validity (aim 3)

Next, we investigated potential associations between self-reported and parent-reported constructs belonging to the same or similar domains. These included inattention, impulsivity or impulsiveness-hyperactivity and inhibitory control, and were correlated with error rates and reaction times in the standardised computer task and the novel CAVE task. To this end, we computer Spearman’s rho correlation coefficients, and applied the FDR method for multiple testing correction.

##### Adults

Firstly, we correlated self-reported measures of inhibition with task performance (error rates and reaction times) and did not find any significant correlations in either of the two tasks. Simiarly, none of the correlations between task performance metrics in the standardised, computer-based task and the novel CAVE task were statistically significant or survived FDR correction. The full correlation matrix, and FDR-corrected and uncorrected *p*-values are presented in full in Table S1 of the Supplementary Materials.

##### Children

Similar to the adult sample, we did not find any significant correlations in either of the two tasks for parent-reported measures relevant to inhibition and task performance, nor between task performance metrics across the two tasks. Nonetheless, we found moderate correlations between metrics within the same task. Speficially, there was a negative correlation between error rates in mixed blocks in the CAVE task and reaction time in Go blocks in the same task (r = − 0.58, p_FDR corrected_ = 0.003, 95% CI [-0.79,-0.24), and a negative correlation between error rate in mixed blocks in the computer task and reaction time in Go blocks in the same task (r = − 0.62, p_FDR corrected_ = 0.001, 95% CI [− 0.80, − 0.31). The full correlation matrix, and FDR-corrected and uncorrected *p*-values are presented in full in Table S2 of the Supplementary Materials.

Exploratory correlations between functional brain activity, behavioural performance and self- and parent-reports are reported in the Supplementary materials for children (Tables S10 and S12), and for adults (Tables S11 and S13).

#### Discriminant validity (aim 3)

Next, we investigated potential associations between self-reported and parent-reported constructs that refer to separate, but related executive functioning domains, including planning and organisation skills, shifting and working memory, and error rates and reaction times in the standardised computer task and the novel CAVE task.

##### Adults

In the adult sample, we found a medium correlation between reaction time in Go blocks in the CAVE task and the Plan/organise subscale of the BRIEF-A (rho = 0.56, p_FDR corrected_ = 0.03, 95% CI [0.17, 0.82]). None of the other correlations were statistically significant or survived FDR correction. The full correlation matrix, and FDR-corrected and uncorrected *p*-values are presented in full in Table S1 of the Supplementary Materials.

##### Children

There were no correlations between task performance metrics and parent-reported constructs that were statistically significant or that survived FDR correction. The full correlation matrix, and FDR-corrected and uncorrected *p*-values are presented in full in Table S2 of the Supplementary Materials.

Exploratory correlations between functional brain activity, behavioural performance and self- and parent-reports are reported in the Supplementary materials for children (Tables S10 and S12), and for adults (Tables S11 and S13).

#### Feasibility (aim 3)

The tasks were feasible for both the adult and the children, as reflected by completion rates (adult sample: 24/24, 100%; children sample: 39/40, 97.5%). Only one participant (age = 4, female) refused to wear the fNIRS cap and attempt the behavioural tasks and hence did not complete the study. All other participants complied with the testing protocol.

#### Acceptability (aim 3)

**Fig. 4:**
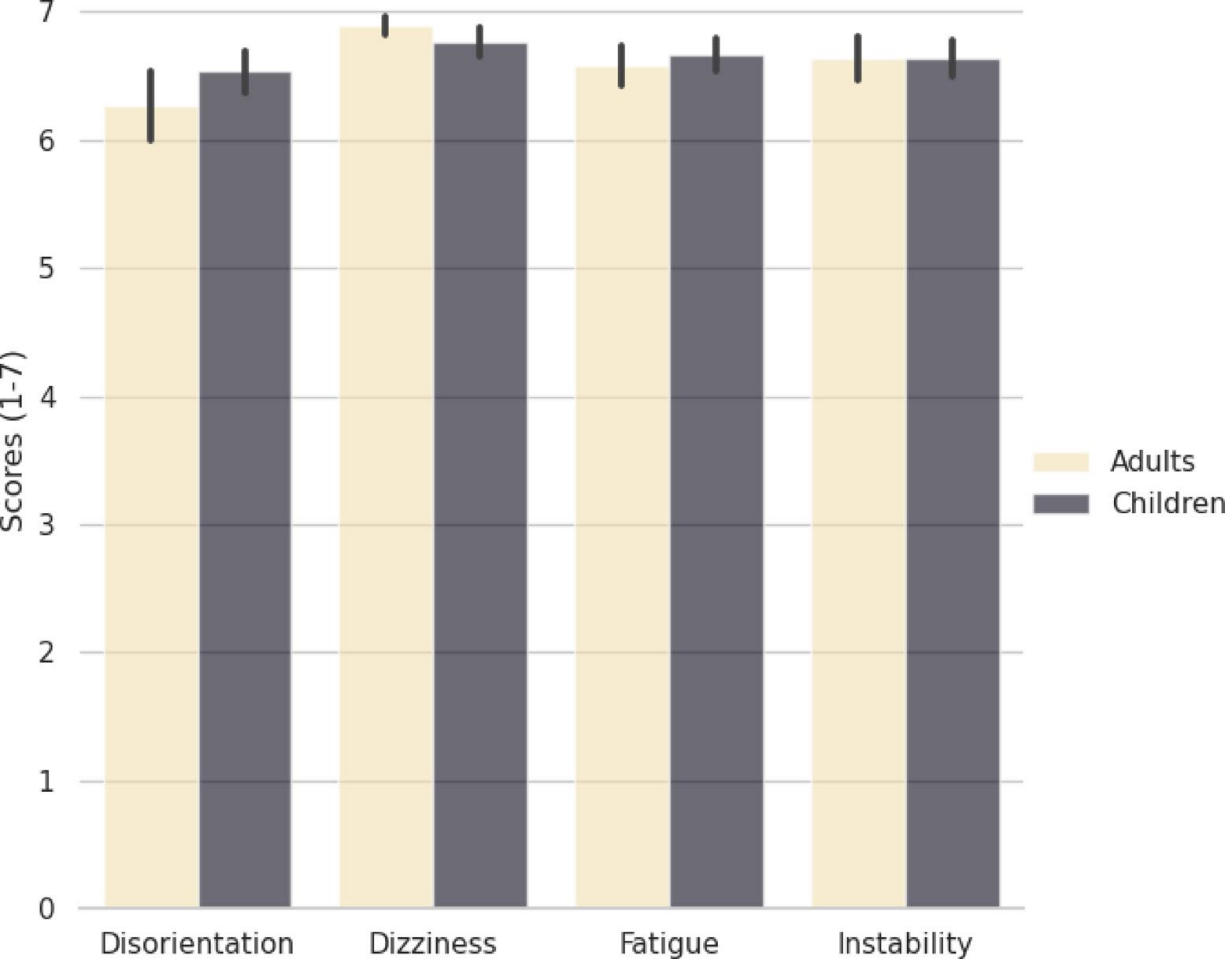
Virtual reality-induced symptoms and effects in children and adults. For children, the VRISE subscale was completed by parents in consultation with their children. Scores ranged from 1 to 7, and higher scores indicated less VR-induced symptoms and effects. Error bars represent the standard error of the mean (SEM).

The novel CAVE task was found to be acceptable for both developmental samples. Most participants reported the absence of VR-induced symptoms and effects or very mild symptoms (see Fig. 4).

We also examined possible associations between VR-induced symptoms and effects and task performance in the two developmental groups. There were no significant associations in either group (see Tables S1 and S2 in the Supplementary Materials for full details).

### fNIRS results

#### Mixed > Go contrast in adults (aim 4)

Our final aim focused on characterising the neural correlates of response inhibition during a 2D and immersive 3D task, and assessing associations between neural activity and behavioural performance. In line with our hypothesis that stronger activation patterns will be found in mixed blocks compared to Go blocks, the analysis of fNIRS data focused on the Mixed > Go contrast of interest. fNIRS data consisted of HbO and HbR AUC values for each channel, and one-sample channel-wise t-tests were run to test if there were significantly larger hemodynamic changes in the Mixed blocks compared to the Go blocks.

**Fig. 5:**
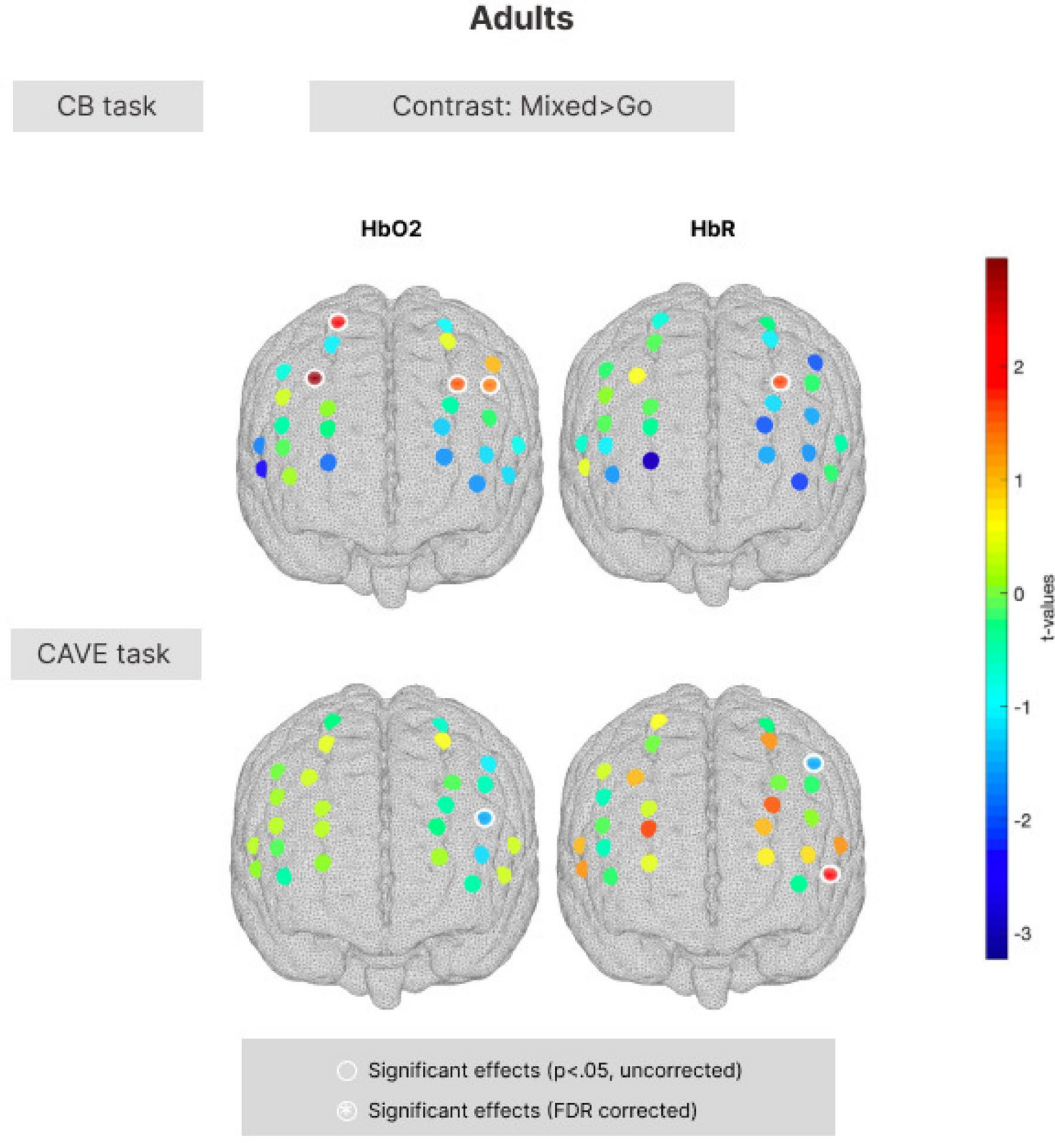
Group-level t-value maps for the Mixed > Go contrast in the standard computer-based task (top) and in the novel CAVE task (bottom) in the adult group. Statistically significant channels (*p* < 0.05, uncorrected) have a white outline. Channels surviving FDR correction for multiple comparisons have a white outline and are marked with a white asterisk. Positive t-values correspond to a HbO_2_ increase and a HbR decrease; negative t-values correspond to a HbO_2_ decrease and a HbR increase.

In the adult sample in the computer-based task, the channels covering the superior frontal gyrus and middle frontal gyrus (channels 9, 13, and 18) showed significant changes in HbO_2_ and HbR (*p* < 0.05) during the mixed blocks of the task. Nonetheless, none of these channels survived FDR correction for multiple comparisons. In the novel CAVE task, the channels covering the middle frontal gyrus and inferior frontal gyrus (channels 16, 19, 24) showed significant changes in HbO_2_ and HbR (*p* < 0.05) during the mixed blocks of the task and similarly did not survive FDR correction. Figure 5 displays brain activation patterns for the adult sample.

#### Functional activity and task performance associations in adults (aim 4)

To further assess associations between fNIRS signal, comprised of HbO and HbR AUC values for each of the channels, and response inhibition at the behavioural level in the adult sample, a multiple regression linear model was specified using the lm function in the *lme4* package in R. fNIRS signal was entered as the outcome, and block type (Go/Mixed blocks), task type (CB/CAVE), chromophores (HbR, HbO_2_), error rates, task type × chromophore and task type × error rate as predictors. Statistically significant interactions were followed-up using pairwise contrasts in the *emmeans* package in R. The analysis focused on the channels with significant changes in HbO_2_ and HbR in the Mixed > Go contrast (FDR corrected and uncorrected). Specifically, channels 9, 13, 16, 18, 19 and 24 were included, covering the superior, middle frontal, and inferior frontal gyri.

The model was significant, *F*(7, 456) = 5.43, *p* < 0.001, explaining 7.7% of the variance (*R*^2^ = 0.077; adjusted *R*^2^ = 0.063). A main effect of task revealed significantly greater activation during the CAVE task compared to the computer-based task (CB; β = 0.00001269, *p* = 0.005). Critically, a significant task type × chromophore interaction (β = − 0.0000172, *p* = 0.003) indicated that this effect was specific to HbO signals. Follow-up contrasts confirmed that CAVE trials elicited significantly greater HbO activation than the computer task trials (t(456) = − 3.65, *p* < 0.001), with no task-related differences in HbR responses. The full regression model is presented in Table 1.

**Table 1:** Multiple regression model with fNIRS signal as the outcome, and block type (Go/Mixed blocks), task type (CB/CAVE), chromophores (HbR, HbO_2_), error rates, task type x chromophore and task type x error rate as predictors in the adult sample.

Predictor	β	Std. Error	p-value
(Intercept)	0.00000159	0.00000512	0.757
Block (Mixed)	− 0.00000189	0.00000349	0.590
Task (CAVE)	0.00001269	0.00000454	0.005**
Chromophore (HbR)	− 0.0000014	0.00000435	0.751
Age	0.00000001	0.00000012	0.943
Error Rate	0.00005339	0.00009574	0.577
Task (CAVE)*Chromophore (HbR)	− 0.0000172	0.00000586	0.003 **
Task (CAVE)*Error Rate	0.0001309	0.0001050	0.213

The analysis focused on the channels with significant changes in HbO_2_ and HbR in the Mixed > Go contrast (FDR corrected and uncorrected). Specifically, channels 9, 13, 16, 18, 19 and 24 were included, covering the superior, middle frontal, and inferior frontal gyri.

To supplement this analysis, Spearman correlations were further ran between task performance measureas (error rates, reaction time) and signal, respectively for self-reports and fNIRS signal in the channels found to be significantly activated in the Mixed > Go contrast before multiple comparison corrections. These are reported in Table S11 and S13 in the Supplementary materials.

#### Mixed > Go contrast in children (aim 4)

**Fig. 6:**
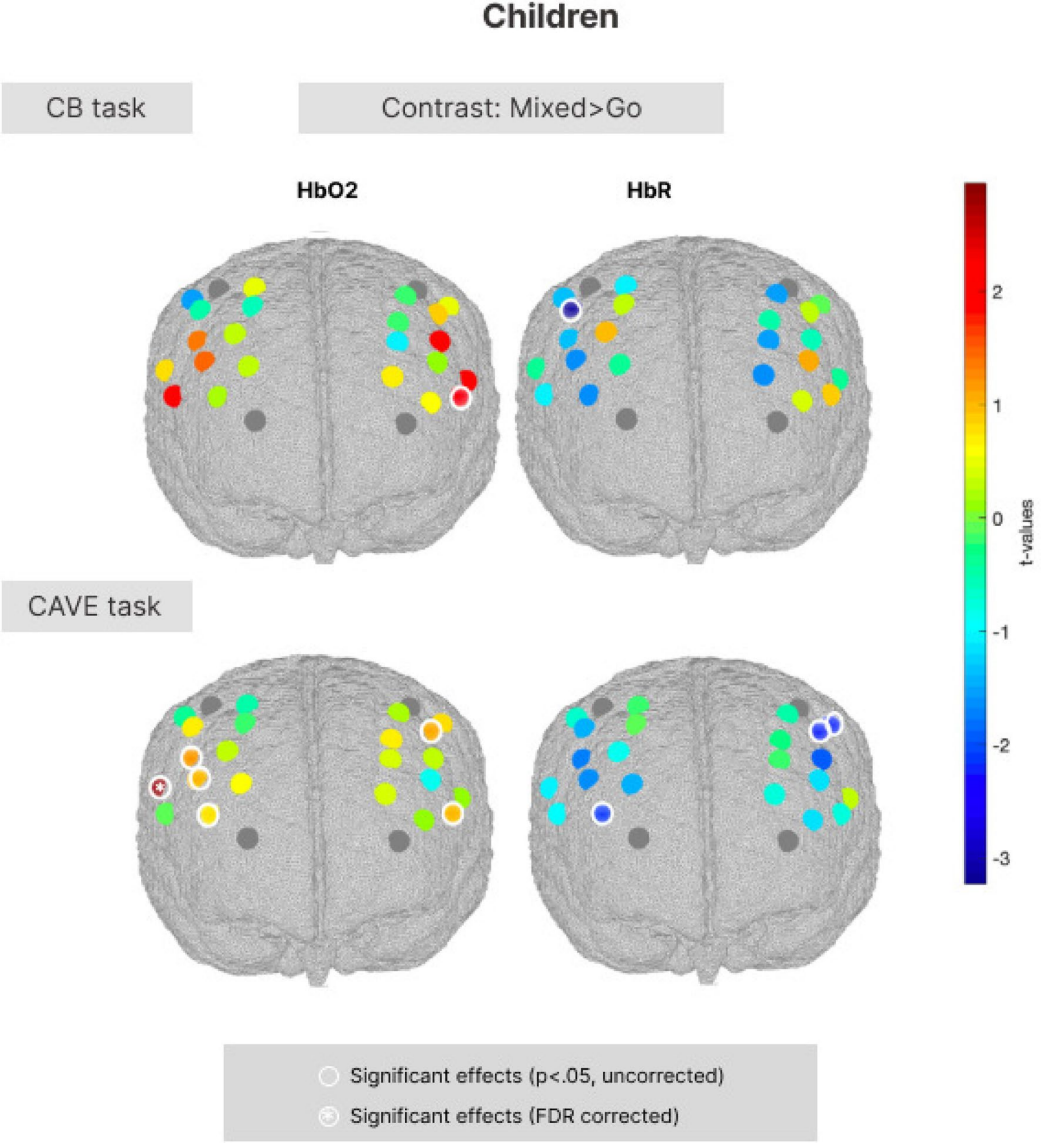
Group-level t-value maps for the Mixed > Go contrast in the standard computer-based task (top) and in the novel CAVE task (bottom) in the early childhood group. Statistically significant channels (*p* < 0.05, uncorrected) have a white outline. Channels surviving FDR correction for multiple comparisons have a white outline and are marked with a white asterisk. Positive t-values correspond to a HbO_2_ increase and a HbR decrease; negative t-values correspond to a HbO_2_ decrease and a HbR increase.

In children in the computer-based task, the channels covering the middle frontal, precentral and inferior frontal gyri (channel 11 and 23) showed significant changes in HbO_2_ and HbR (*p* < 0.05) during the mixed blocks of the task. Nonetheless, none of these channels survived FDR correction for multiple comparisons. In the novel CAVE task, we found significant changes in HbO_2_ and HbR (*p* < 0.05) during the mixed blocks in channels covering the middle frontal, precentral and inferior frontal gyri (channels 2, 6, 7, 9, 14, 15, 23), but only the channel corresponding to the inferior frontal gyrus (channel 9), survived FDR correction for multiple comparisons. Figure 6 displays brain activation patterns for the early childhood sample.

#### Functional activity and task performance associations in children (aim 4)

To assess associations between fNIRS signal and response inhibition at the behavioural level in the early childhood sample, the same multiple linear regression model was run, and any significant interaction terms were followed up using pairwise contrasts. This time, the analysis focused on the channels with significant changes in HbO_2_ and HbR in the Mixed > Go contrast (FDR corrected and uncorrected). Specifically, channels 2, 6, 7, 9, 14, 15, and 23 were included, covering the middle frontal, precentral and inferior frontal gyri.

The overall model was significant, *F*(7, 1568) = 8.25, *p* < 0.001, explaining 4% of variance (*R*^2^ = 0.04; adjusted *R*^2^ = 0.03). The main effect of error rate was statistically significant, indicating that higher error rates were associated with increased neural activity (β = − 0.00007466, *p* = 0.014). The interaction between task and error rate also reached significance (β = 0.0001574, *p* < 0.001), indicating that error rate was more strongly associated with increased fNIRS activation in the CAVE task compared to the computer-based task. This pattern was supported by follow-up contrasts showing significantly greater activation for the CAVE than the computer task trials at average error rate (0.159; t(1568) = − 2.99, *p* = 0.003), suggesting increased neural engagement in the CAVE under conditions of reduced accuracy. The full regression model is presented in Table 2.

**Table 2:** Multiple regression model with fNIRS signal as the outcome, and block type (Go/Mixed blocks), task type (CB/CAVE), chromophores (HbR, HbO_2_), error rates, task type x chromophore and task type x error rate as predictors in the children sample.

Predictor	β	Std. error	*p*-value
(Intercept)	0.00000936	0.00001111	0.400
Block (Mixed)	0.00000183	0.00000522	0.726
Task (CAVE)	− 0.00000558	0.00000787	0.479
Chromophore (HbR)	− 0.00000520	0.00000645	0.420
Age	− 0.00000124	0.00000201	0.538
Error Rate*	− 0.00007466	0.00003031	0.014*
Task (CAVE)*Chromophore (HbR)	− 0.00000925	0.00000903	0.306
Task (CAVE)*Error Rate	0.0001574	0.00003336	< 0.001***

The analysis focused on the channels with significant changes in HbO_2_ and HbR in the Mixed > Go contrast (FDR corrected and uncorrected). Specifically, channels 2, 6, 7, 9, 14, 15, and 23 were included, covering the middle frontal, precentral and inferior frontal gyri.

To supplement this analysis, Spearman correlations were further ran between task performance measureas (error rates, reaction time) and signal, respectively for parent-reports and fNIRS signal in the channels found to be significantly activated in the Mixed > Go contrast before multiple comparison corrections. These are reported in Table S10 and S12 in the Supplementary materials.

## Discussion

This is the first study to investigate the brain correlates of response inhibition during a naturalistic task in early childhood by integrating age-appropriate functional near-infrared spectroscopy (fNIRS) imaging with an immersive cave automatic virtual environment (CAVE). To this end, we developed a novel response inhibition task in a CAVE for children and compared it with a standardised age-appropriate response inhibition task whilst simultaneously recording functional brain activity from the bilateral frontal cortices, areas associated with response inhibition^5,34,35^. The novel and standardised tasks were first administered in a sample of adults to ensure they are successful in measuring the construct of interest, and to investigate the feasibility and acceptability of the newly developed task before being used in an early childhood sample (3–7-year-olds). Overall, participants in both groups displayed increased error rates in mixed blocks compared with Go blocks across the novel and standardised tasks, and, in general, displayed poorer task performance in the immersive task. The novel CAVE task was feasible and acceptable to both participant groups, as indicated by high completion rates and no or minimal adverse VR-induced symptoms or effects. The functional brain activity pattern indicated higher functional activation in mixed blocks compared with Go blocks in the left inferior frontal gyrus in the novel CAVE task, and this was associated with higher error rates. These findings were specific to the children sample and indicate that the novel CAVE task was sensitive to age differences. Implications for multimodal naturalistic setups in paediatric samples are discussed.

### Task performance

Our first aim was to compare behavioural performance in a computerised Go/No-Go task with that in a naturalistic Go/No-Go task in a CAVE in two samples of adults and young children. In line with our hypothesis, task performance results in both developmental groups were consistent with typical performance in Go/No-Go tasks. Go/No-Go tasks are characterised by the repeated execution of a motor response to Go stimuli and withholding responses to No-Go stimuli. The frequency of each trial type can be manipulated experimentally, but Go stimuli are predominant such that there is a prepotency of responding^10^. Due to the predominance of Go stimuli, these tasks are characterised by higher error rates in Mixed blocks, during which No-Go stimuli are introduced. Lower accuracy in mixed blocks was confirmed in both samples in the current study in the CAVE task. Furthermore, reaction time distributions are also usually positively skewed^50^, as was the case in the current study. While performance on such tasks is established for traditional computer-based tasks, it is less clear how participants’ performance on standardised tasks compares with performance in an immersive VR scenario.

In our study, participants in both samples displayed increased error rates in the novel CAVE task, compared with the 2D computer task, but the pattern differed by developmental group. Specifically, adults displayed worse performance during the Go blocks whilst children had lower accuracy in the mixed blocks of the CAVE. This aligns with Bailey et al.^28^, the only other study to date that compared a VR task with a 2D equivalent to investigate inhibitory control in young children aged 4–6 years. Comparable to our results, they found that children performed worse on their inhibitory control measure in the immersive VR condition compared with children who completed the same task on a 2D screen. Comparing reaction time in the two tasks, children responded uniformy across the two paradigms but the adult group took considerably longer to make their response to Go stimuli in the immersive environment. This finding is similar to a study in adults comparing performance between VR, 3D and 2D tasks, where they reported longer reaction times in the VR and 3D conditions, together with a longer period of time fixating features of the paradigms^51^. Furthermore, computational models of the speed-accuracy trade-off propose that reaction time in each trial reflects the time required for the nervous system to encode a stimulus, make a decision and then execute the motor response^52^. In our CAVE task, responses were made through hand gestures, which require hand–eye coordination and specific motor strategies, possibly making them less familiar than traditional human–computer interaction modelities such as keyboards and mice^53^. It is also possible that the content of the virtual scene introduced cognitive load, leading to longer reaction times^54^. Notably, this effect was isolated to adults, who might have have been less accustomed to virtual environments than children. Nonetheless, we did not record prior exposure to virtual environments in either sample.

Furthermore, Bailey et al. did not report reaction times in their VR and 2D inhibition tasks, and no previous research to our knowledge has compared time to respond in VR environments across developmental groups^28^. In general, it is worth noting that previous studies have observed longer reaction times in more complex environments, whilst others did not find any differences^55^, and therefore future research on this topic is essential, including accounting for prior exposure or familiarity with immersive technologies.

### Developmental differences

The second aim was to establish if the tasks were suitable for capturing developmental differences in inhibitory control. Contrary to our hypothesis that age would be positively associated with performance in the CAVE task, these associations did not survive multiple comparison correction. Since we report low to moderate effect sizes and the study was powered to detect large effects, it is possible that our sample was not adequately powered to detect these age-related associations. Despite this, the age-appropriateness of the tasks can be indirectly evidenced through the increased neural activity in the children group in relation to higher error rates, especially in the more naturalistic, immersive task. Moreover, though the tasks were developed specifically for children, we observed the same pattern of behavioural performance in adults, albeit with lower error rates overall, denoting decreased, age-related difficulty. Compared with adults, children generally had poorer performance across all metrics, with the exception of reaction time in the CAVE, as discussed above. These findings likely reflect the prolonged developmental trajectory of inhibitory control, which continues to develop in early childhood through adolescence and early adulthood^3^, suggesting the tasks were successful in capturing neurodevelopmental differences. In the CAVE, higher error rates could have been driven, at least in part, by the visual novelty of the stimuli presented in the immersive setting and the richness of the environment surrounding them, which was designed to be salient for children (i.e., playground). In fact, previous research has found the novelty effect of immersive VR learning environments to impact performance until adaptation takes place^56,57^. Interestingly, this effect did not dissipate entirely following adaptation through a tutorial^56^, perhaps indicating that longer exposure is necessary for full adaptation to occur. This would be a plausible explanation for our study, as all participants first completed practice trials in both tasks, and were allowed time to familiarise themselves with the virtual environment. Furthermore, children’s knowledge that Go and No-Go stimuli in the computer task were not real, combined with the narrow field of view of the computer screen in the standardised computer task which might have facilitated better inhibition, resulting in better performance and therefore could partly explain the absence of differences between Go and mixed blocks in the computer task.

### Psychometric properties

The third aim of this study was to understand the psychometric properties of the novel CAVE task. Our findings on convergent and discriminant validity provide a nuanced perspective on the psychometric properties of the novel CAVE task in comparison to traditional computer-based assessments. For convergent validity, our study found no significant correlations between self-reported (adults) or parent-reported (children) measures of inhibition and task performance metrics in either the CAVE or the standardised computer task. Crucially, there were also no statistically significant correlations observed between task performance metrics (error rates and reaction times) when directly comparing the computer-based task and the novel CAVE task in either developmental group. This suggests that the two tasks were not equivalent. However, within the same task for children, moderate negative correlations were found between error rates in mixed blocks and reaction times in Go blocks in both the CAVE (r = − 0.58) and computer task (r = − 0.62). These findings present a point of divergence from a recent systematic review of naturalistic tasks, including those using virtual environments, assessing inhibitory control, which found that most studies comparing the VR task with a standardised equivalent reported significant correlations, albeit ranging from negligible to high (r = 0.03–0.82)^16^. Nonetheless, our results are on par with Bailey et al.^28^, who reported poor convergent validity and found children performed better in the standard 2D task compared to the 3D version. These mixed findings could reflect the qualitative differences between the two environments, and could be in part influenced by the design^58^. Unlike the studies included in the review using HMDs, the current study used an immersive CAVE. CAVEs have been shown to provide a more natural sense of embodiment and provide more movement freedom for participants, and therefore our CAVE setup could have been experienced differently^59,60^. Nonetheless, it is worth noting that few studies directly compared CAVEs and HMDs, and those that did report mixed results, possibly reflecting the rapid advancement of VR technologies, and of HMDs specificially^61^. On discriminant validity, the overlap between CAVE task performance and planning skills in adults in the current study aligns with the findings of the review of naturalistic inhibitory control tasks^16^. The review noted that few studies formally assessed discriminant validity, and those that did often reported mixed or poor results, supporting the notion that naturalistic tasks may tap into a broader range of cognitive processes^16^.

### User experience

Besides psychometric properties, we were also interested in understanding user experience in the form of feasibility and acceptability. Both adults and children successfully completed the testing procedures and complied with wearing the hardware necessary for the study (fNIRS system, 3D-shutter glasses and motion tracking glove). Furthermore, participants generally did not experience any VR symptoms or had very mild symptoms. Importantly, this work shows that VR is safe, feasible and acceptable in children as young as 3 years old and can be used for cognitive assessment. This is important, because VR research in paediatric populations has mainly focused on its use as a distraction intervention to reduce pain and anxiety in medical settings^62,63^, and, to our knowledge, only two other studies to date have used VR in healthy young children^28,29^. In older children and adolescents, several studies employing VR tasks have been successfully conducted in the context of ADHD^64–66^. In a next step, future studies may consider extending the use of virtual reality paradigms to younger children with conditions characterised by deficits in inhibition, for whom naturalistic metods might facilitate engagement.

### Neural activity using fNIRS

Our final aim was to characterise the neural correlates of response inhibition in 2D and 3D scenes, and explore associations between neural activity and behavioural performance. In both tasks in children, we found significant changes in HbO_2_ and HbR in the middle frontal, precentral and inferior frontal gyri; however, none of these channels survived multiple comparison correction in the standard computer task. Other fNIRS studies using non-immersive Go/No-Go tasks in paediatric populations with age ranges similar to ours reported increased functional activation in the right middle and inferior frontal gyri and in the bilateral supramarginal gyri^32^, recruitment of the right dorsolateral prefrontal cortex in children aged 4–10^67^, and 7–12 years^30^, and increased functional activation in the right middle and inferior frontal hyri and bilateral supramarginal gyri in 4 to 7-year-olds^32^. These findings partly align with ours in terms of the channels preferentialy recruited in these tasks, though ours did not withstand the Benjamini–Hochberg correction^68^. It is agreed that inhibitory control tasks activate a network consisting of bilateral mesial, medial, inferior frontal and parietal cortices, and therefore it is not surprising that there are slight differences in the regions identified in different studies, within the inhibitory control network umbrella^6^. For instance, a meta-analysis of eleven studies employing Go/No-Go tasks during fMRI found that tasks using simpler stimuli demonstrated distinct patterns of concurrence compared with those using more complex stimuli^13^. Specifically, they found differential right-laterialised prefrontal-parietal circuits only in the complex tasks, requiring involvement from related, but distinct executive functions such as working memory. The definition of “simple” Go/No-Go tasks, those in which the No-Go stimulus–response association remains the same, captures the computer task used in our study and could help explain the weaker signal captured in this task.

The only channel that survived multiple comparisons correction corresponded to the left inferior frontal gyrus and this was the case only in the novel CAVE task and only in the children sample. These findings are consistent with previous research using fMRI to investigate the shared inhibitory neurocognitive network^35^, showing more frequent left lateralisation in Go/No-Go tasks specifically^69,70^. This left frontal lateralisation has been further documented in several studies conducted in developmental samples^71,72^. Furthermore, evidence from neurological patients with brain lesions indicate that the integrity of the IFG in particular is critical for successful implementation of response inhibition over motor responses^34^. The IFG is also used to characterise areas of absolute diversity for executive functioning^35^.

One possible explanation for this result is the complexity of the CAVE task relative to the computerised task, placing higher strain on cognitive control to attend to the task at hand and ignore task-irrelevant information in the immersive environment, and possibly requiring more recruitment of the inhibitory network. Increased cognitive load in immersive virtual environments has been reported in previous studies comparing 3D and 2D tasks^73^, and immersive 3D surgical procedures with conventional setups^74^. Furthermore, previous research has found different mechanisms to be implicated in immersive and non-immersive encovironments, even when performance was otherwise comparable. In^73^, participants completing the VR condition using HMDs had lower performance than those in the computer-based condition, and showed greater reliance on explicit cognitive mechanisms, rather than implicit. The increased functional activation in the IFG in the CAVE task only could also be related to an increased prepotency of response due to the involvement of the whole arm and torso movement compared to a simple key tap, which could place higher strain on inhibitory processes. This is in line with previous studies showing that increased cognitive load is related to tasks requiring more complex motor skills^74^. Nonetheless, since this is the first study to use a VR-fNIRS protocol to study response inhibition in children, there are no direct comparisons with neural correlates we can make with equivalent studies.

None of the channels in the frontal regions survived multiple comparison correction in the adult sample. This is in contrast to prior studies employing a VR-fNIRS setup in adults, reporting increased functional activation in the VR paradigms compared with non-VR equivalents^75,76^, as do studies using a combined VR and fMRI protocol^77^. However, a key distinction is that the VR and computer paradigms used in the current study were specifically designed to be age-appropriate for young children aged 3–7 years, and therefore the cognitive demand of the tasks on response inhibition was likely low, as reflected by the low error rates in the behavioural data. Nonetheless, when examining associations between neural and behavioural data in adults, we found increased functional activation during trials of the CAVE task, suggesting that engaging the body more actively has implications for responding, and implicitly for the recruitment of the inhibitory control network. Moreover, as is standard in fNIRS research, neural activation was indexed by increases in oxyhemoglobin (HbO₂)^78^.

### Strengths, limitations and recommendations for future work

This work has several strengths. First, it is the first study to develop and validate an age-appropriate response inhibition task for young children in an immersive CAVE, whilst simultaneously recording neuroimaging data using wearable fNIRS. Second, the task was tested in a sample of adults first, to ensure the setup was feasible and acceptable, and then further validated in a sample of 3–7-year-olds. It is also important to note that the novel task was compared with a similar computer-based Go/No-Go task to test its convergent validity, and with parent reports of other cognitive domains to assess discriminant validity. Third, for the first time in young children we used short separation channels and showed that superficial signal contamination is present in children and adults. Notably, we demonstrated that the localisation of neural activity can be improved using superficial signal regression in both tasks, but to a larger extent in the novel CAVE task. The in-depth method, results and implications are discussed at length in a separate paper^79^.

Nonetheless, building and testing a naturalistic paradigm presents multiple challenges and there are limitations that should be acknowledged, as well as several directions for further studies that we would like to highlight. While the novel naturalistic task was compared with an equivalent computerised Go/No-Go task, the two tasks used different stimuli, motor responses and hence were not identical. This could have impacted the comparisons we can draw between the two tasks. However, with this study using a within-subjects design, the fact that the tasks were not identical might have helped improve user experience and reduce boredom. It is also worth noting that while VR-induced symptoms and effects were absent or mild for most participants, future studies using VR in young children should further enquire about familiarity with technology, any prior experience with 2D and 3D media and consider including motion sickness as potential confounds^80^. Furthermore, our study focused on typically developing children. Children were recruited from our centre’s database, and those with neurodevelopmental diagnoses or a family history of such diagnoses were not eligible for the study. Nonetheless, it is worth noting that, due to resource and time constraints, we were not able to conduct clinical interviews to confirm the absence of such diagnoses. Relatedly, the results presented here cannot be extrapolated to specific diagnoses, and, to our knowledge, no studies to date have used VR to assess response inhibition in young children with neurodevelopmental or psychiatric diagnoses^16^. Nonetheless, the evidence we have thus far from children with ADHD aged 6–14 years indicates that this methodology is suitable for assessing inhibitory processes^25,64,81^. It is also important to acknowledge that here we used a CAVE system. Whilst it may have advantages such as increased awareness of one’s body in space, a wider viewing angle, less restricted movement and higher screen resolution^61^, it is also resource intensive and therefore not readily accessible. Having shown that the CAVE is feasible and acceptable to children as young as 3-years-old, future studies can consider similar paradigms with head-mounted displays, which are considerably more accessible and have the potential to be used remotely^16,82^. Furthermore, another limitation of this work related to statistical power and multiple comparison correction in the fNIRS data. While we conducted an a priori power analysis using a medium to large effect size at an alpha threshold of 0.05 with 80% power, this was based on the effect reported in a meta-analysis of fNIRS studies assessing multiple constructs (not only inhibitory control) in a range of different ages (3–17 years). Furthermore, this effect size referred to task performance rather than brain activity data, and as such the study might not have been adequately powered for fNIRS analysis. For this reason, we report both uncorrected and corrected results but focus our discussion on data collected from channels that passed the *p* < 0.05 threshold, corrected.

It is also important to note that dynamic tasks can elicit systemic changes^83^, and therefore future studies should consider adding additional monitors of systemic physiology such as electrocardiograms, blood pressure or respiration besides short channels^84^ to further improve robustness of the fNIRS signals. In addition, motion tracking could be used and averaged data included as a predictor in models examining associations between functional activity and behavioural performance.In the current study, we chose not to add these due to the challenges of using multiple pieces of equipment on young children. Nonetheless, now that we have shown this setup to be feasible, future research may consider taking a Systemic Physiology Augmented fNIRS (SPA-fNIRS) approach^85^. Finally, as described in the Methods section, the localisations of the optodes and channels were recorded individually for each participant, but co-registered on a common MRI template. This is common practice in fNIRS research and a compromise between accuracy, cost^86^ and using a more age-appropriate, naturalistic method to measure brain activity^43^.

## Conclusion

To summarise, this is the first study to investigate response inhibition in young children in naturalistic settings (a CAVE) and concomitantly measure its neural correlates during unrestricted movement. Here we demonstrate that a novel CAVE task is a valid measure of response inhibition, activating frontal brain regions identified as part of the neural inhibitory control network in prior neuroimaging research. We further show that an immersive VR and fNIRS setup can be safely, feasibly and acceptably be used in children as young as 3 years old. Our work further opens multiple avenues for future research. For example, the task could be further expanded to include several levels of difficulty, and could be integrated with other physiological measures, such as eye-tracking. It may also be useful for assessing response inhibition in conditions characterised by deficits in response inhibition, such as ADHD.

## Methods

### Pre-registration

The protocol for this study was submitted and pre-registered on the Open Science Framework (https://osf.io/wyp4s/).

### Ethical approval

Ethical approval was granted by the Birkbeck Ethics Committee (2021072), and all methods were performed in accordance with the relevant guidelines and regulations. Informed consent was obtained from all participants and/or their parents or legal guardians.

### Sample size

We conducted an *a priori* power calculation in G* power (Erdfelder et al., 1996) based on a medium to large correlation (r = 0.39) or a large difference (d = 0.83) between two groups at an alpha threshold of 0.05 with 80% power which resulted in 30 participants per group (see Figure S1 in Supplementary Materials). It should be noted, however, that the effect size is based on a meta-analysis aggregating results of fNIRS studies assessing multiple constructs (including executive functions and inhibitory control specifically) in children and adolescents (aged 3–17 years) as research using fNIRS in early childhood is limited^87^. Nonetheless, this large difference between the two groups is further supported by a previous study using a TV and a VR condition to measure inhibitory control in younger children (N = 52, 26 children in each condition)^28^. In that study, children aged 4–6 years demonstrated better inhibitory control in the TV condition than in the VR condition (Cohen’s *d* = 0.89; TV condition: *M* = 0.83, *SD* = 0.15; VR condition: *M* = 0.67, *SD* = 0.21), even after controlling for age. Whilst this is a behavioural study without fNIRS, to our knowledge it is the only study investigating a VR inhibition task with a comparator in young children. Finally, the estimated sample size is also comparable to those reported in studies using fNIRS to study response inhibition using standard Go/No-Go tasks in developmental samples [e.g., 22 children aged 4–6 years^88^, 17 children aged 4–8^37^, 21 children aged 4–10 years^89^, 19 children with a mean age of 6 years^90^.

### Participants

Twenty-four healthy adults (range: 18–59 years, M_age_ = 30.38, SD = 10.54, 58.3% female) and thirty-nine healthy children (range: 3–7 years, M_age_ = 4.45, SD = 1.08, 35.9% female) were enrolled into the study. For recruitment, a two-pronged approach was used: adults were recruited via institutional advertisements and word-of-mouth, and children were recruited from the Centre for Brain and Cognitive Development participant database. Participants were not eligible to participate if they had a neurodevelopmental, or psychiatric/physical health condition, or a family history of neurodevelopmental or psychiatric disorders. For adults, this was established based on self-report and scores on Part 1 of the Adult ADHD Self-Report Scale (ASRS) (scores >14 might be indicative of ADHD and were excluded). For children, this was based on information held in the database and parent reports. We applied multiple exclusion criteria for behavioural, and fNIRS data analysis respectively. For the behavioural analysis, three children were excluded because their overall performance was less than 50% on the computer-based (N = 2), and on the novel CAVE inhibition tasks (N = 2). One child was excluded from both the computer-based and the CAVE task (hence why only 3 children were excluded) (M_age_ = 4.5, SD = 0.71, 1 female). None of the adult participants were excluded based on task performance. Consequently, 24 adults (M_age_ = 30.38, SD = 10.54, 58.3% female) and 36 children (M_age_ = 4.44, SD = 1.11, 36.1% female) were included in the behavioural analysis. For the fNIRS analysis, 11 participants were excluded from the CAVE task analysis because of poor fNIRS data (4 adults, and 7 children) and 6 participants were excluded from the computer-based task for poor fNIRS data quality (4 adults, 2 children). Criteria for exclusion is detailed in the ‘fNIRS data analysis’ section below. The final fNIRS analytical sample included 30 children for the CAVE task (M_age_ = 4.5 years, SD = 1.14, 21 males), 30 children for the computer-based task (M_age_ = 4.53 years, SD = 1.14, 21 males), 20 adults participants for the CAVE task (M_age_ = 31.3 years, SD = 11.33, 10 males) and 21 adult participants for the computer-based task (M_age_ = 30.95 years, SD = 11.16, 10 males).

### Procedure

Participants or their parents (for children) were e-mailed an information sheet and consent form ahead of the testing session. Prior to the testing session, participants (or their parents) were asked to complete a short online questionnaire asking for basic demographic information (age, gender) and self-report/parent questionnaires measuring attention, impulsivity and hyperactivity symptoms. For adults, these questionnaires were the Adult Self-Report ADHD Scale, and the Barrett Impulsiveness Scale; for children, parents were asked to complete the Strengths and Weaknesses of Attention Deficit/Hyperactivity Disorder Symptoms and Normal Behavior Scale. Following this, participants and their parents attended a 1-hour testing session at the new ToddlerLab at Birkbeck, in central London. Upon arrival, participants and their parents were presented with the information sheet and consent form again. Then, two block-designed tasks were evaluated for each participant, in counterbalanced order: (1) a validated computerised inhibitory control task (Go/No-Go)^47^; (2) a novel CAVE inhibitory control task (Go/No-Go). Participants wore a wireless fNIRS system during both tasks, and for the novel CAVE task participants also wore custom 3D-printed shutter-glasses which enabled active-stereo viewing to facilitate immersion into the virtual space, and a glove fitted with motion tracking markers which allowed them to interact with the virtual objects in the virtual environment. The glove was always worn on the right hand.

### Computerised inhibitory control task

**Fig. 7:**
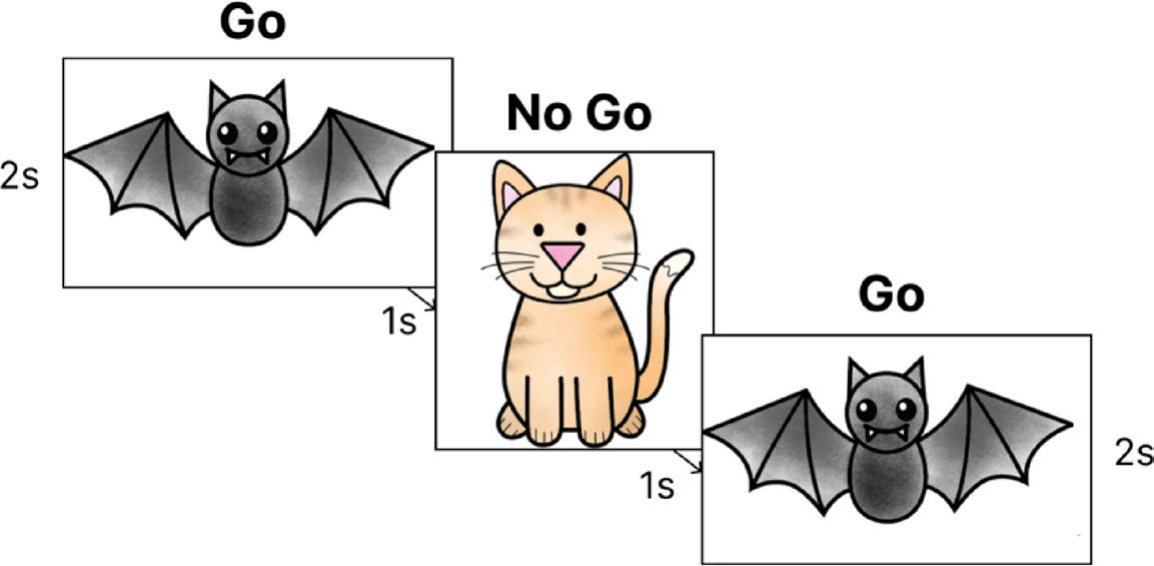
Example Go and No-Go trials from the standardised computer-based response inhibition task.

We used a standardised child-friendly version of a Go/No-Go task^47^, in which participants were presented with static illustrations of either bats or cats on a laptop screen. Participants were told their role is to help protect a town by catching the bats, because they could turn into vampires, and protect the cats. To ‘catch’ the bats, participants pressed the space bar on the laptop keyboard. To ensure participants understood the instructions, the task started with two practice trials consisting of a bat (Go trial) and a cat (No-Go trial). The task consisted of 120 trials, split into 90 Go trials and 30 No-Go trials, and followed a block design, with 12 blocks split into 6 Go-only blocks and 6 Go/No-Go blocks (mixed blocks) (50% Go, 50% Mixed blocks). The blocks were alternated, and the task always started with a Go block. Mixed blocks had 10 trials each, and Go blocks had 11, 9, 10, 9, 11 and 10 trials. Each stimulus was presented on screen for 2 s, with a 1-s inter-stimulus interval. Participants were presented with a fixation cross between trials and the inter-block time varied between 8 and 12-s and was randomised (Fig. 7). The task took between 6 And 8 min to complete, and participants wore a portable fNIRS system throughout.

### CAVE inhibitory control task

**Fig. 8:**
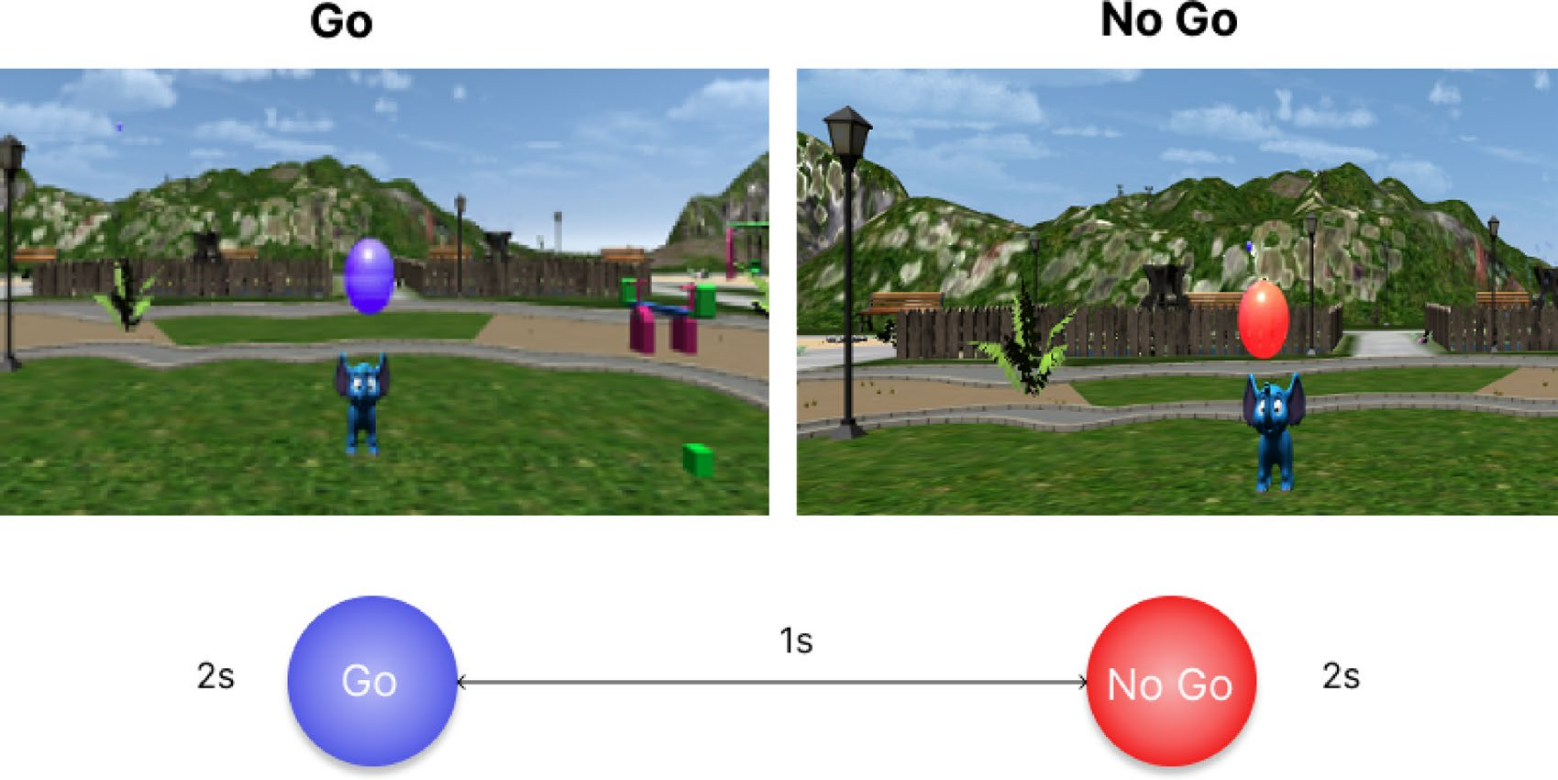
Example Go and No-Go trials from the novel CAVE response inhibition task.

Response inhibition in the VR environment consisted of an adapted version of the computer-based task. The number, length and order of the trials and blocks were the same. Instead of the cats and bats, the VR task involved the popping of bubbles coming out of an elephant’s trunk. Participants were presented with bubbles of two different colours. They were instructed to pop the blue bubbles (Go trials) and refrain from popping the red bubbles (No-Go trials). Participants interacted with the bubbles via the motion tracking gloves that tracked their hand’s movements. Each stimulus was presented on screen for 2 seconds, with a 1 second interstimulus interval (Fig. 8). Participants wore the portable fNIRS system throughout the task and custom 3D-printed shutter glasses with head motion tracking markers, and motion tracking gloves.

To mimic the fixation cross in the computer-based task, participants were asked to fixate a star on the elephant’s head. The star appeared at the start of the task and between the blocks. The inter-block, interstimulus and stimulus presentation times were the same as in the computer task. The same outcome variables were derived from this task.

### Cave automatic virtual environment (CAVE) setup

**Fig. 9:**
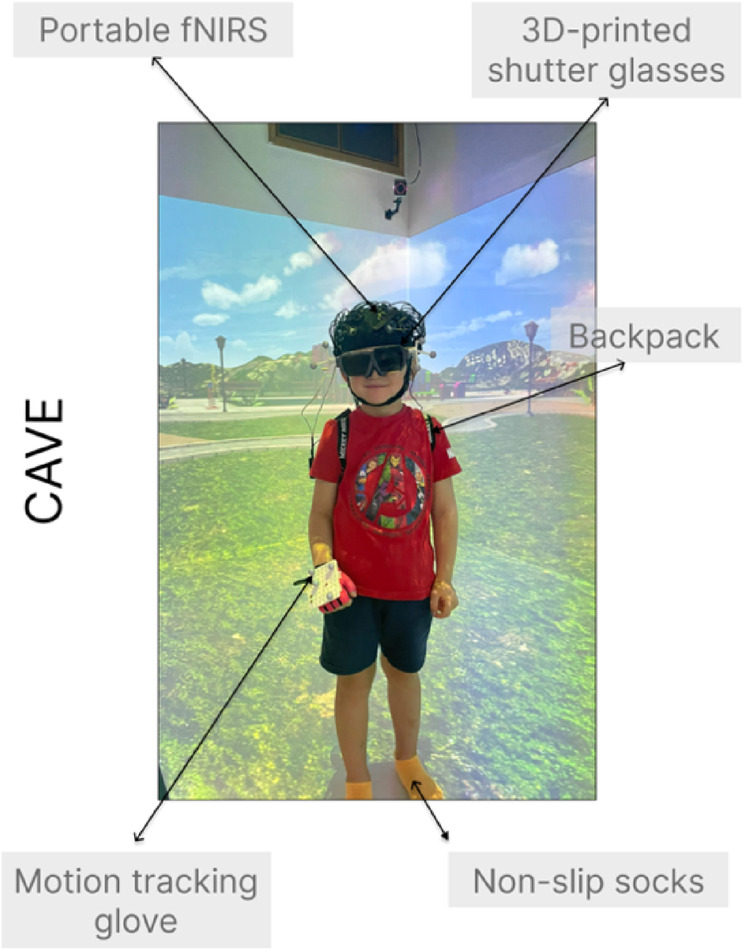
Experimental setup in the CAVE. Participants wore a portable fNIRS system, consisting of two continuous wave fNIRS devices (Brite MKII, Artinis Medical Systems BV, Netherlands) mounted on a neoprene cap, a backpack for tucking away the fNIRS system’s wires, a motion tracking glove for interacting with the virtual objects, custom-made 3D-printed shutter glasses allowing 3D vision, and non-slip socks to ensure safe and unrestricted in the CAVE.

The CAVE system used here (Mechdyne Corporation) is part of the Birkbeck ToddlerLab in London. The four-sided custom-designed display system involves one frontal (4.3 × 2m) and two side projection walls (2.4 × 2m each), as well as a projection floor (4.3 × 2m). The front wall and the floor use two overlapped and blended single chip laser projectors, each having a resolution of 2716 × 1528 pixels (total resolution: 3297 × 1528 pixels). The side walls are served by a single laser projector (2716 × 1528 pixels). To interact with the CAVE, participants wore custom 3D-printed child/adult-sized liquid crystal display (LCD) shutter glasses, enabling active stereo viewing which allowed full immersion into the CAVE. To enable the orientation and rotation of the virtual scenes as participants were moving in the CAVE, the LCD shutter glasses had head motion tracking markers attached on the sides. To interact with the virtual scenes (i.e., for bubble popping), motion tracking markers were also attached to the glove participants were wearing on their right hand (Fig. 9). These markers were tracked by the four six-degrees-of-freedom optical motion tracking cameras located in each of the four corners of the CAVE (Vero 1.3 X, Vicon).

### fNIRS acquisition and pre-processing

**Fig. 10:**
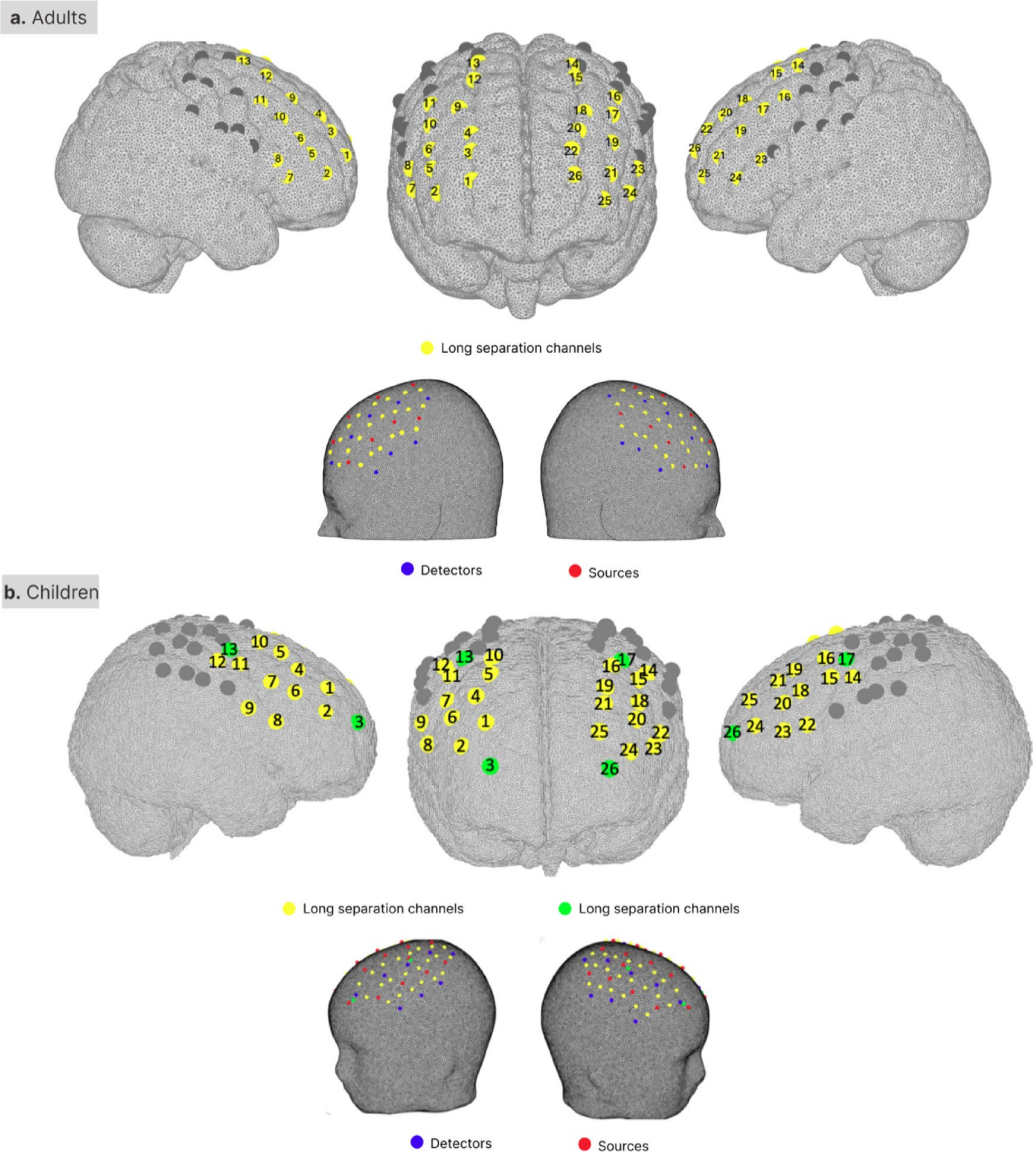
Optodes’ arrangement and corresponding channel numbers. Long separation channels are marked in yellow, and short separation channels are marked in green. Channels that were not included in the analysis are depicted in grey.

Two wireless continuous wave Brite MKII fNIRS devices (Artinis Medical Systems BV, the Netherlands) were combined onto the same cap to measure the cortical changes in HbO_2_ and HbR from the dorsolateral prefrontal cortex and the motor cortex bilaterally during the CB and CAVE tasks. Each system had 10 light sources and 8 detectors. Light sources emitted light at 760 nm and 840 nm, and the sampling frequency of the acquisition was set to 25 Hz. Optodes were arranged to form 44 long separation channels with a source-detector distance of 3 cm for adults and 2.5 cm for children. We have further modified the children’s array to include 4 additional short separation channels (source-detector distance = 1 cm). Recommendations on the use of superficial signal regression to improve the localisation of functional brain activity in mobile children and more details on the pre-processing and analysis of fNIRS data can be found elsewhere^84^. The placement of the optodes and their corresponding channel number is shown in Fig. 10a for adult participants and in Fig. 10b for children. Because we were interested in areas associated with response inhibition, the analysis focused on the 26 channels covering the bilateral dorsolateral prefrontal cortex^5,30,35^.

To achieve reliable placement for participants, we aligned the cap to the Fp1 and Fp2 landmarks on the 10 to 20 electrode placement system, and recorded frontal videos of the cap placement for each participant which were used for co-registration on a common template. The localisations of the optodes and channels were co-registered onto a 5-year-old MRI template from the Neurodevelopmental MRI Database of the University of South Carolina^91^. To this end, the MRI template was 3D-printed to create a head model and find the ideal placement of the cap, including the head model coordinates of the sources and detectors and the anatomical landmarks (Nasion, Inion, Cz, right and left preauricular points, Fp1, Fp2, Fpz, F7, F8, O1, and O2). The anatomical landmarks were registered using a 3D magnetic digitizer (FasTrak, Polhemus, Colhester, Vermont, United States). In a next step, these data and the frontal videos of the participants were inputted into STORM-NET (https://github.com/yoterel/STORM-Net) to estimate the position of the optodes and landmarks^92^. To identify the anatomical locations of the channels, we used the LONI Probabilistic Brain Atlas (LPBA40)^93^. The specific channel assignments for each region of interest are included in the Supplementary Materials (Table S5).

#### Self-report measures

The **Barratt Impulsiveness Scale** (BIS^94^) is used extensively in the field of impulsivity and is the gold-standard self-report questionnaire for this construct. The BIS has 30 items and consists of six correlated subdomains: attention (“I concentrate easily”), cognitive instability (“I have racing thoughts”), motor impulsiveness (“I buy things on impulse”), perseverance (“I change jobs”), cognitive complexity (“I save regularly”) and self-control (“I plan tasks carefully”). The six subdomains can be further grouped into three second-order factors in accordance with Barratt’s three-factor theory^94^: attentional impulsiveness (attention, cognitive instability), motor impulsiveness (motor, perseverance) and non-planning impulsiveness (cognitive complexity, self-control). The BIS contains 30 items which are rated on a scale of 1 (rarely/never) to 4 (almost always). Some items are reverse scored (items 1,7,8,9,10,12,13,15, 20, 29, 30). Scores range from 30 to 120, with higher scores indicating higher impulsivity. The scale shows excellent internal reliability (Cronbach’s alpha = 0.83).

The 18-item **Adult ADHD Self-Report Scale** (ASRS)^95^ was used to assess ADHD symptomology. The scale is formed of two parts – part A (questions 1–6), which has been found to be the most predictive of adult ADHD symptoms, and part B (questions 7–18), which provides insight into symptom frequency^95^. Although the ASRS can be scored in multiple ways, the 0–24 scoring method has been shown to outperform the 0–4 scoring^96–98^. This method was therefore used, and part A items were ranked quantitatively with scores ranging from 0 to 4, for a maximum possible score of 24.

The **Behaviour Rating Inventory of Executive Function—Adult Version** (BRIEF-A) is a 75-item standardised self-report or informant measure assessing executive functions or self-regulation in adults aged 18–90 years (Roth et al., 2005). The scale produces an overall score (Global Executive Composite) and two indexes, metacognition and behavioural regulation. The behavioural regulation index is formed of four scales (Inhibit, Shift, Emotional Control, and Self-Monitor), while the metacognition index has five scales (Initiate, Working Memory, Plan/Organise, Task Monitor, and Organisation of Materials). The scale also includes three validity scales (negativity, inconsistency, and infrequency). The items are rated on a 3-point frequency scale (0–3; never-often). The scale shows good internal reliability for the self-report scales (Cronbach’s alpha = 0.73-0.90) and for the indexes and the Global Executive Composite (Cronbach’s alpha = 0.93–0.96). Higher scores indicate worse executive functioning.

The **Strengths and Weaknesses of ADHD-symptom and Normal-behaviour** (SWAN) Scale is an 18-item parent questionnaire assessing inattention and hyperactivity-impulsivity in children. The scale is based on the symptom criteria outlined in the Diagnostic and Statistical Manual of Mental Disorders, Fourth Edition (DSM-IV)^99^ and Fifth Edition (DSM-V)^100^. Items are worded positively (e.g. “Remembers daily activities), relative to normal behaviour expectations rather than with a focus on deficits. Each item is scored on a 7-point scale, ranging from -3 (far below average) to + 3 (far above average), and measures strengths and weaknesses on one continuum. The scale has shown excellent internal validity (Cronbach’s alpha = 0.88) and results in normally distributed data in the general population^101^. Furthermore, the scale has been shown to be highly sensitive and specific in distinguishing between children with and without ADHD and other psychiatric disorders^102^. Higher scores indicate greater symptomology.

The **Behaviour Rating Inventory of Executive Function – Preschool Version** (BRIEF-P) is a 63-item standardised informant measure assessing executive functions or self-regulation in pre-school children aged 2 to 5 years and 11 months^103^. Informants can be parents, teachers or other habitual child caregivers. Similarly to the BRIEF-A, the BRIEF-P produces an overall score (Global Executive Composite), three indexes (inhibitory self-control, flexibility and emergent metacognition), and five clinical scales (inhibition, emotional control, flexibility, working memory and plan/organise). The BRIEF-P also includes two validity scales (inconsistency and negativity). The measure takes between 10 and 15 min to administer, and questions are answered on a 3-point frequency scale (0–3; never-often). The scale shows good internal reliability (Cronbach’s alpha = 0.80-0.95 for parents and 0.90-0.97 for teachers) and moderate test–retest reliability (0.78-0.90 for parents and 0.64-0.94 for teachers). Higher scores indicate worse executive functioning.

The VR-induced symptoms and effects (VRISE) subdomain of the **Virtual Reality Neuroscience Questionnaire** (VRNQ^104^) was used to evaluate the acceptability of the CAVE. Items were answered on a 7-point Likert scale, ranging from 1 (extremely low) to 7 (extremely high). Higher scores on the domain indicated a more positive outcome. The domain took approximately 1 to 2 min to administer. The VRNQ scale demonstrated good convergent and discriminant validity, as well as good construct validity for the VR induced symptoms and effects subscale (alpha = 0.89). Due to a technical problem, participants were presented with only 4 of the 5 items of the VRISE subdomain, such that they were not asked about symptoms of nausea. The 4 items that participants or their parents were presented with enquired about disorientation, dizziness, and fatigue during the novel CAVE task.

### Data analysis

#### Behavioural data analysis

Demographic characteristics are presented for the participants included in the analyses for the adult and the early childhood groups. The feasibility and acceptability of the task are also assessed. Some of the most frequently used indictors of feasibility are completions rates, inconvenience and reasons for non-completion^105^. Acceptability refers to the appropriateness of the task, based on anticipated or experienced responses to the task^106^. Enquiring about VR-induced symptoms and effects is one method to evaluate acceptability of virtual environments^107^. Before any statistical analysis, data were checked for normality by visual inspection of histograms and using the Shapiro–Wilk normality test, recommended for small sample size (< 50 participants). Reaction time distributions are usually positively skewed^50^. In our data, we found that reaction time in Go blocks in the CAVE task was non-normally distributed for adults (Shapiro–Wilk = 0.90, *p* = 0.04) and reaction time in Go blocks in the computer task was non-normally distributed for children (Shapiro–Wilk = 0.93, *p* = 0.03). Furthermore, error rates in Go blocks in the CAVE task were non-normally distributed for both adults (Shapiro–Wilk = 0.69, *p* < 0.001) and children (Shapiro–Wilk = 0.83, *p* < 0.001), as were error rates in mixed blocks in adults (Shapiro–Wilk = 0.75, *p* < 0.001). Error rates in Go (adults: Shapiro–Wilk = 0.24, *p* < 0.001; children: Shapiro–Wilk = 0.73, *p* < 0.001) and mixed blocks (adults: Shapiro–Wilk = 0.76, *p* < 0.001; children: Shapiro–Wilk = 0.88, *p* = 0.001) in the computerised task were also non-normally distributed for both groups.

Paired student t-tests were used for within-group comparisons for normally distributed variables, and Wilcoxon ranked tests were used for variables with skewed distributions. For between-group comparisons, we used independent student t-tests or Mann–Whitney U tests respectively. To assess task performance, we compared task performance outcomes (error rates, reaction times) across the two tasks in the adult and the early childhood groups separately. To compute 95% confidence intervals (Cis), we applied a bootstrapping procedure. Specifically, 10,000 bootstrap samples were generated by resampling the observed differences with replacement. The 95% CI were determined as the 25^th^ and 97.5^th^ percentiles of the distribution of bootstrapped medians. This approach is non-parametric and does not assume normality of the data.

Moreover, we were also interested to check if the tasks are suitable for capturing developmental differences in response inhibition and compared task performance outcomes between the two developmental groups. To validate the novel CAVE task, we were interested to check convergent validity by assessing correlations between the CAVE task performance measures and task performance on the standardised computer task, as well as on self- or parent-reported constructs belonging to the same or similar domains, including inattention, impulsivity or impulsiveness-hyperactivity and inhibitory control. Finally, we assessed discriminant validity through correlations between the CAVE task performance measures and self- or parent-reported constructs that relate to separate, but related executive functioning domains, including planning and organisation skills, shifting and working memory.

Self- or parent-reported scale scores were assessed for normality by visually inspecting histograms and using the Shapiro–Wilk test. In the early childhood sample, SWAN (Shapiro–Wilk = 0.83, *p* < 0.001), VRISE (Shapiro–Wilk = 0.61, *p* < 0.001) and the BRIEF-P subscale assessing Shifting (Shapiro–Wilk = 0.89, *p* = 0.01) were not normally distributed. SWAN scores were left skewed, with most participants having higher scores indicative of higher symptomology. VRISE scores were similarly left skewed, as most participants did not experience any VR-induced symptoms and effects. Finally, the distribution of the Shift subscale was right skewed. In the adult group, VRISE (Shapiro–Wilk = 0.75, *p* < 0.001) and all subscales derived from BRIEF-A except the Shift subscale were non-normally distributed, namely Emotional Control (Shapiro–Wilk = 0.87, *p* = 0.012), Inhibition (Shapiro–Wilk = 0.84, *p* = 0.004), Plan/Organise (Shapiro–Wilk = 0.91, *p* = 0.043), and Working Memory (Shapiro–Wilk = 0.89, *p* = 0.031). Therefore, we used pairwise Spearman’s correlations to assess convergent and discriminant validity, removing missing datapoints, and applied the False Discovery Rate (FDR; Benjamini & Hochberg, 1995) method to correct for multiple comparisons.

#### Outlier correction

All behavioural data were screened for outliers. For non-normally distributed variables (assessed using the Shapiro–Wilk test), we used the interquartile range (IQR) approach, identifying values falling below the 2.5th percentile or above the 97.5th percentile. This is more robust to non-parametric data. Outliers were then replaced with the median value for the respective variable to reduce the impact of extreme values while preserving the overall distribution. For parametric data, outliers were defined as values exceeding ± 3 standard deviations from the mean and were replaced with the variable’s mean^108^.

In the adult sample, eight outliers from seven different participants were identified M_age_ = 34.13 years, SD_age_ = 12.99; 4 male), pertaining to the VRISE, emotional control, inhibition, shift and working memory subscales of the BRIEF-A, error rate in Go blocks in the computer task, and reaction time in the computer task. In the children, seven outliers from six different participants were identified (M_age_ = 4.71 years, SD_age_ = 0.95; 4 male), and related to SWAN scores, error rates in Go blocks in both tasks, error rates in mixed blocks in the computer task and reaction time in the computer task.

#### fNIRS data analysis

To process the fNIRS data, we followed the procedure described in Pinti et al. (2024). First, noisy channels, such as those with no clear heart rate peak or with detector saturation or considerable motion artifacts were excluded after visually inspecting the raw intensity fNIRS data. The intensity signals were further processed using Homer 2^109^. There were several steps to the analysis pipeline. First, raw fNIRS data were converted to changes in optical density (*hmrIntensity2OD*), and a wavelet-based algorithm was used to correct for motion artifacts (iqr = 0.8 for children, iqr = 1.5 for adults, *hmrMotionCorrectWavelet*)^110^. Next, we applied a band-pass filter (Fc = [0.01, 0.1] Hz; *hmrBandpassFilt*) to optical density signals, which were then converted into changes in HbO_2_ and HbR using the modified Beer-Lambert law (DPF = [5.5 4.7] for the children, DPF = [6 6] for adults; *hmrOD2Conc*^111^. Following these steps, participants were excluded from analysis if they had less than 50% good quality fNIRS channels, and less than 3 blocks with performance > 50% for the Go and Mixed blocks. Details on excluded participants for either poor fNIRS data quality or poor performance in the behavioural tasks were provided in the ‘Participants’ subsection above. In addition, Table S14 in the Supplementary Materials shows the number of Go and No-Go trials included in the analysis for each block type, each task and each developmental group. Before any statistical analysis, data were checked for normality using the kstest function in Matlab to run the Kolmogorov–Smirnov test for each channel. Data were normally distributed and we used a general linear model-based deconvolution approach to estimate the hemodynamic response separately for the Go and Mixed blocks. This was done for each participant, channel and chromophore. For the children, this included the regression of the short channel with the highest correlation to each long separation channel^112^ (trange = [− 2 32], glmSolveMethod = 1, idxBasis = 1, paramsBasis = [1.5 1.5], rhoSD_ssThresh = 1.5 cm, flagSSmethod = 1, driftOrder = 0, flagMotionCorrect = 0; *hmrDeconvHRF_DriftSS*). The area under the curve (AUC) for the Go and Mixed blocks for both the novel CAVE and the standardised computer task was calculated within a time window from 15 to 25 s for children and 10 to 20 s for adults following the start of each task, to include the largest changes in the response. The decision to choose different time windows for adults and children was guided by previous research showing that the peak latency of the haemodynamic response function is delayed in younger populations^113,114^, and that the time-to-peak decreases with age^115^. The AUC was calculated for both HbO_2_ and HbR for each participant and then used in the group-level analyses. To test whether there were significant larger hemodynamic changes in the Mixed blocks compared to the Go blocks, we ran one-sample channel-wise t-tests. The False Discovery Rate (FDR) method was used to correct for multiple comparisons^68^.

Exploratory subgroup analyses were performed in high impulsive participants (as determined using a score of > 72 on the BIS in adults and using a median split in the SWAN for children as no clinically relevant cut-offs exist^116^, and are reported in Tables S3 and S4 in the Supplementary materials.

## Electronic supplementary material

Below is the link to the electronic supplementary material.

Supplementary Material 1

## Data Availability

The data supporting this manuscript can be made available upon request to the corresponding author through a formal data sharing and project affiliation agreement.

## References

[1] Feola, B. et al. Overlapping and unique brain responses to cognitive and response inhibition. *Brain Cogn.* **166**, 105958 (2023). 36796257 10.1016/j.bandc.2023.105958 PMC11186579

[2] Mostofsky, S. H. & Simmonds, D. J. Response inhibition and response selection: Two sides of the same coin. *J. Cogn. Neurosci.* **20**, 751–761 (2008). 18201122 10.1162/jocn.2008.20500

[3] Best, J. R. & Miller, P. H. A developmental perspective on executive function. *Child Dev.* **81**, 1641–1660 (2010). 21077853 10.1111/j.1467-8624.2010.01499.x PMC3058827

[4] Whedon, M. Developmental changes in electrophysiology and speech during problem-solving as predictors of inhibitory control in preschool. No Pagination Specified (Human Development and Family Studies, US, 2020).

[5] Zhou, X. et al. Inhibitory control in children 4–10 years of age: Evidence from functional near-infrared spectroscopy task-based observations. *Front. Hum. Neurosci.* **15**, 798358 (2022). 35046786 10.3389/fnhum.2021.798358 PMC8762317

[6] Hung, Y., Gaillard, S. L., Yarmak, P. & Arsalidou, M. Dissociations of cognitive inhibition, response inhibition, and emotional interference: Voxelwise ALE meta-analyses of fMRI studies. *Hum. Brain Mapp.* **39**, 4065–4082 (2018). 29923271 10.1002/hbm.24232 PMC6866358

[7] Munakata, Y. et al. A unified framework for inhibitory control. *Trends Cogn. Sci.* **15**, 453–459 (2011). 21889391 10.1016/j.tics.2011.07.011 PMC3189388

[8] Ferguson, H. J., Brunsdon, V. E. A. & Bradford, E. E. F. The developmental trajectories of executive function from adolescence to old age. *Sci. Rep.* **11**, 1382 (2021). 33446798 10.1038/s41598-020-80866-1 PMC7809200

[9] Roth, R. M., Isquith, P. K. & Gioia, G. A. Assessment of executive functioning using the behavior rating inventory of executive function (BRIEF). In *Handbook of executive functioning* (eds. Goldstein, S. & Naglieri, J. A.) 301–331 (Springer, New York, NY, 2014). 10.1007/978-1-4614-8106-5_18.

[10] Littman, R. & Takács, Á. Do all inhibitions act alike? A study of go/no-go and stop-signal paradigms. *PLoS ONE* **12**, e0186774 (2017). 29065184 10.1371/journal.pone.0186774 PMC5655479

[11] Verbruggen, F. et al. A consensus guide to capturing the ability to inhibit actions and impulsive behaviors in the stop-signal task. *Elife* **8**, e46323 (2019). 31033438 10.7554/eLife.46323 PMC6533084

[12] White, C. N., Ratcliff, R. & Starns, J. J. Diffusion models of the flanker task: Discrete versus gradual attentional selection. *Cognit. Psychol.* **63**, 210–238 (2011). 21964663 10.1016/j.cogpsych.2011.08.001 PMC3195995

[13] Simmonds, D. J., Pekar, J. J. & Mostofsky, S. H. Meta-analysis of Go/No-go tasks demonstrating that fMRI activation associated with response inhibition is task-dependent. *Neuropsychologia* **46**, 224–232 (2008). 17850833 10.1016/j.neuropsychologia.2007.07.015 PMC2327217

[14] Bezdjian, S., Baker, L. A., Lozano, D. I. & Raine, A. Assessing inattention and impulsivity in children during the Go/NoGo task. *Br. J. Dev. Psychol.* **27**, 365–383 (2009). 19812711 10.1348/026151008X314919 PMC2757760

[15] Aliko, S., Huang, J., Gheorghiu, F., Meliss, S. & Skipper, J. I. A naturalistic neuroimaging database for understanding the brain using ecological stimuli. *Sci. Data* **7**, 347 (2020). 33051448 10.1038/s41597-020-00680-2 PMC7555491

[16] Dina, L.-M., Smith, T. J., Hauser, T. U. & Dommett, E. J. Naturalistic assessments across the lifespan: Systematic review of inhibition measures in ecological settings. *Neurosci. Biobehav. Rev.* **167**, 105915 (2024). 39395771 10.1016/j.neubiorev.2024.105915 PMC11870848

[17] Nastase, S. A., Goldstein, A. & Hasson, U. Keep it real: rethinking the primacy of experimental control in cognitive neuroscience. *Neuroimage* **222**, 117254 (2020). 32800992 10.1016/j.neuroimage.2020.117254 PMC7789034

[18] Vigliocco, G. et al. Ecological brain: Reframing the study of human behaviour and cognition. Preprint at 10.31234/osf.io/zr4nm (2023). 10.1098/rsos.240762 PMC11544371 39525361

[19] Neguț, A., Matu, S.-A., Sava, F. A. & David, D. Virtual reality measures in neuropsychological assessment: A meta-analytic review. *Clin. Neuropsychol.* **30**, 165–184 (2016). 26923937 10.1080/13854046.2016.1144793

[20] Van der Elst, W., Van Boxtel, M. P. J., Van Breukelen, G. J. P. & Jolles, J. A large-scale cross-sectional and longitudinal study into the ecological validity of neuropsychological test measures in neurologically intact people. *Arch. Clin. Neuropsychol.* **23**, 787–800 (2008). 18930628 10.1016/j.acn.2008.09.002

[21] Allen, K. et al. Using games to understand the mind. *Nat. Hum. Behav.* **8**, 1035–1043 (2024). 38907029 10.1038/s41562-024-01878-9

[22] Borgnis, F. et al. Available virtual reality-based tools for executive functions: A systematic review. *Front. Psychol.* **13**, 833136 (2022). 35478738 10.3389/fpsyg.2022.833136 PMC9036486

[23] Nolin, P. et al. ClinicaVR: Classroom-CPT: A virtual reality tool for assessing attention and inhibition in children and adolescents. *Comput. Hum. Behav.* **59**, 327–333 (2016).

[24] Rizzo, A. A. et al. The virtual classroom: A virtual reality environment for the assessment and rehabilitation of attention deficits. *Cyberpsychol. Behav.* **3**, 483–499 (2000).

[25] Parsons, T. D., Bowerly, T., Buckwalter, J. G. & Rizzo, A. A. A controlled clinical comparison of attention performance in children with ADHD in a virtual reality classroom compared to standard neuropsychological methods. *Child Neuropsychol. J. Norm. Abnorm. Dev. Child. Adolesc.* **13**, 363–381 (2007). 10.1080/13825580600943473 17564852

[26] Pollak, Y. et al. The utility of a continuous performance test embedded in virtual reality in measuring ADHD-related deficits. *J. Dev. Behav. Pediatr. JDBP* **30**, 2–6 (2009). 19194324 10.1097/DBP.0b013e3181969b22

[27] Bailey, J. O. & Bailenson, J. N. Immersive virtual reality and the developing child. In *Cognitive development in digital contexts* 181–200 (Elsevier, 2017). 10.1016/B978-0-12-809481-5.00009-2.

[28] Bailey, J. O., Bailenson, J. N., Obradovic, J. & Aguiar, N. R. Virtual reality’s effect on children’s inhibitory control, social compliance, and sharing. *J. Appl. Dev. Psychol.*10.1016/j.appdev.2019.101052 (2019).

[29] Bulgarelli, C., Pinti, P., Aburumman, N. & Jones, E. J. H. Combining wearable fNIRS and immersive virtual reality to study preschoolers’ social development: A proof-of-principle study on preschoolers’ social preference. *Oxf. Open Neurosci.* **2**, 012 (2023). 10.1093/oons/kvad012 PMC10913823 38596237

[30] Zhong, X., Wang, C., Xu, M., Yuan, X. & Jiang, C. Physical training improves inhibitory control in children aged 7–12 years: An fNIRS study. *Behav. Brain Res.* **463**, 114902 (2024). 38341102 10.1016/j.bbr.2024.114902

[31] Kerr-German, A., Namuth, A., Santosa, H., Buss, A. T. & White, S. To snack or not to snack: Using fNIRS to link inhibitory control to functional connectivity in the toddler brain. *Dev. Sci.* **25**, e13229 (2022). 35005833 10.1111/desc.13229 PMC9232869

[32] McKay, C., Wijeakumar, S., Rafetseder, E. & Shing, Y. L. Disentangling age and schooling effects on inhibitory control development: An fNIRS investigation. *Dev. Sci.* **25**, e13205 (2022). 34865293 10.1111/desc.13205

[33] Fiske, A. et al. The neural correlates of inhibitory control in 10-month-old infants: A functional near-infrared spectroscopy study. *Neuroimage* **257**, 119241 (2022). 35537598 10.1016/j.neuroimage.2022.119241 PMC7616317

[34] Swick, D., Ashley, V. & Turken, A. U. Left inferior frontal gyrus is critical for response inhibition. *BMC Neurosci.* **9**, 102 (2008). 18939997 10.1186/1471-2202-9-102 PMC2588614

[35] Saylik, R., Williams, A. L., Murphy, R. A. & Szameitat, A. J. Characterising the unity and diversity of executive functions in a within-subject fMRI study. *Sci. Rep.* **12**, 8182 (2022). 35581269 10.1038/s41598-022-11433-z PMC9114123

[36] Liu, Q., Zhu, X., Ziegler, A. & Shi, J. The effects of inhibitory control training for preschoolers on reasoning ability and neural activity. *Sci. Rep.* **5**, 14200 (2015). 26395158 10.1038/srep14200 PMC4585799

[37] Anderson, A. A. et al. Prefrontal cortex hemodynamics and age: A pilot study using functional near infrared spectroscopy in children. *Front. Neurosci.* **8**, 393 (2014). 25565935 10.3389/fnins.2014.00393 PMC4266015

[38] Lezak, M. D. et al. *Neuropsychological assessment* (Oxford University Press, 2012).

[39] Combe, T., Chardonnet, J.-R., Merienne, F. & Ovtcharova, J. CAVE and HMD: Distance perception comparative study. *Virtual Real.* 1–11 (2023). 10.1007/s10055-023-00787-y. 10.1007/s10055-023-00787-y PMC10054200 37360808

[40] Ghinea, M., Frunză, D., Chardonnet, J.-R., Merienne, F. & Kemeny, A. Perception of absolute distances within different visualization systems: HMD and CAVE. In *Augmented reality, virtual reality, and computer graphics* (eds. De Paolis, L. T. & Bourdot, P.) 148–161 (Springer International Publishing, Cham, 2018). 10.1007/978-3-319-95270-3_10.

[41] Marsh, W. E., Chardonnet, J.-R. & Merienne, F. Virtual distance estimation in a CAVE. In *spatial cognition IX* (eds. Freksa, C., Nebel, B., Hegarty, M. & Barkowsky, T.) 354–369 (Springer International Publishing, Cham, 2014). 10.1007/978-3-319-11215-2_25.

[42] Kaufmann *et al.* Immersive Horizons: Navigating the Impacts of Virtual Reality on Children and Families. In *Children and screens: A handbook on digital media and child and adolescent development, health, and well-being* (Springer Nature, in press).

[43] Pinti, P. et al. A review on the use of wearable functional near-infrared spectroscopy in naturalistic environments. *Jpn. Psychol. Res.* **60**, 347–373 (2018). 30643322 10.1111/jpr.12206 PMC6329605

[44] Scholkmann, F. et al. A review on continuous wave functional near-infrared spectroscopy and imaging instrumentation and methodology. *Neuroimage* **85**(Pt 1), 6–27 (2014). 23684868 10.1016/j.neuroimage.2013.05.004

[45] Gervain, J. et al. Near-infrared spectroscopy: A report from the McDonnell infant methodology consortium. *Dev. Cogn. Neurosci.* **1**, 22–46 (2011). 22436417 10.1016/j.dcn.2010.07.004 PMC6987576

[46] Hartley, C. A. How do natural environments shape adaptive cognition across the lifespan?. *Trends Cogn. Sci.* **26**(12), 1029–1030 (2022). 36272935 10.1016/j.tics.2022.10.002

[47] Schröer, L., Cooper, R. P. & Mareschal, D. Science with Duplo: Multilevel goal management in preschoolers’ toy house constructions. *J. Exp. Child Psychol.* **206**, 105067 (2021). 33610884 10.1016/j.jecp.2020.105067

[48] Maruo, Y. & Masaki, H. A possibility of error-related processing contamination in the No-go N2: The effect of partial-error trials on response inhibition processing. *Eur. J. Neurosci.* **55**, 1934–1946 (2022). 35343617 10.1111/ejn.15658 PMC9324169

[49] Maasalo, K., Lindblom, J., Kiviruusu, O., Santalahti, P. & Aronen, E. T. Longitudinal associations between inhibitory control and externalizing and internalizing symptoms in school-aged children. *Dev. Psychopathol.* **33**, 843–855 (2021). 32662373 10.1017/S0954579420000176

[50] Marmolejo-Ramos, F., Cousineau, D., Benites, L. & Maehara, R. On the efficacy of procedures to normalize Ex-Gaussian distributions. *Front. Psychol.* **5**, 1548 (2015). 25709588 10.3389/fpsyg.2014.01548 PMC4285694

[51] Barrett, R. C. A. et al. Comparing virtual reality, desktop-based 3D, and 2D versions of a category learning experiment. *PLoS ONE* **17**, e0275119 (2022). 36201546 10.1371/journal.pone.0275119 PMC9536585

[52] Myers, C. E., Interian, A. & Moustafa, A. A. A practical introduction to using the drift diffusion model of decision-making in cognitive psychology, neuroscience, and health sciences. *Front. Psychol.* **13**, 1039172 (2022). 36571016 10.3389/fpsyg.2022.1039172 PMC9784241

[53] Arif, S. M. U., Brizzi, M., Carli, M. & Battisti, F. Human reaction time in a mixed reality environment. *Front. Neurosci.* **16**, 897240 (2022). 36061612 10.3389/fnins.2022.897240 PMC9437458

[54] Polechoński, J. & Langer, A. Assessment of the relevance and reliability of reaction time tests performed in immersive virtual reality by mixed martial arts fighters. *Sensors* **22**, 4762 (2022). 35808260 10.3390/s22134762 PMC9268816

[55] Vahle, N. M., Unger, S. & Tomasik, M. J. Reaction time-based cognitive assessments in virtual reality - A feasibility study with an age diverse sample. *Stud. Health Technol. Inform.* **283**, 139–145 (2021). 34545829 10.3233/SHTI210552

[56] Miguel-Alonso, I., Checa, D., Guillen-Sanz, H. & Bustillo, A. Evaluation of the novelty effect in immersive virtual reality learning experiences. *Virtual Real.* **28**, 27 (2024).

[57] Kargut, K., Gutwin, C. & Cockburn, A. Effects of device environment and information layout on spatial memory and performance in VR selection tasks. In *Proceedings of the 2024 CHI conference on human factors in computing systems* 1–17 (Association for Computing Machinery, New York, NY, USA, 2024). 10.1145/3613904.3642486.

[58] Angra, S. et al. Twenty-two years of advancements in augmented and virtual reality: A bibliometric and systematic review. *Front. Comput. Sci.* **7**, 1470038 (2025).

[59] Landowska, A., Royle, S., Eachus, P. & Roberts, D. Testing the potential of combining functional near-infrared spectroscopy with different virtual reality displays—Oculus rift and oCtAVE. In *Augmented reality and virtual reality: Empowering human, place and business* (eds. Jung, T. & tom Dieck, M. C.) 309–321 (Springer International Publishing, Cham, 2018). 10.1007/978-3-319-64027-3_21.

[60] Philpot, A., Glancy, M., Passmore, P. J., Wood, A. & Fields, B. User Experience of Panoramic Video in CAVE-like and Head Mounted Display Viewing Conditions. In *Proceedings of the 2017 ACM international conference on interactive experiences for TV and online video* 65–75 (Association for Computing Machinery, New York, NY, USA, 2017). 10.1145/3077548.3077550.

[61] Peng, K. et al. iVR-fNIRS: Studying brain functions in a fully immersive virtual environment. *Neurophotonics* **11**, 020601 (2024). 38577629 10.1117/1.NPh.11.2.020601 PMC10993907

[62] Bernaerts, S. et al. Virtual reality for distraction and relaxation in a pediatric hospital setting: An interventional study with a mixed-methods design. *Front. Digit. Health* **4**, 866119 (2022). 35712230 10.3389/fdgth.2022.866119 PMC9192964

[63] Eijlers, R. et al. Systematic review and meta-analysis of virtual reality in pediatrics: Effects on pain and anxiety. *Anesth. Analg.* **129**, 1344–1353 (2019). 31136330 10.1213/ANE.0000000000004165 PMC6791566

[64] Mangalmurti, A. et al. Using virtual reality to define the mechanisms linking symptoms with cognitive deficits in attention deficit hyperactivity disorder. *Sci. Rep.* **10**, 529 (2020). 31953449 10.1038/s41598-019-56936-4 PMC6969149

[65] Muhlberger, A. et al. The influence of methylphenidate on hyperactivity and attention deficits in children With ADHD: A virtual classroom test. *J. Atten. Disord.* **24**, 277–289 (2020). 27178061 10.1177/1087054716647480

[66] Camacho-Conde, J. A. & Climent, G. Attentional profile of adolescents with ADHD in virtual-reality dual execution tasks: A pilot study. *Appl. Neuropsychol. Child* **11**, 81–90 (2022). 32400210 10.1080/21622965.2020.1760103

[67] Zhou, X. et al. Inhibitory control in children 4–10 years of age: Evidence from functional near-infrared spectroscopy task-based observations. *Front. Hum. Neurosci.* **15**, 798358 (2021). 35046786 10.3389/fnhum.2021.798358 PMC8762317

[68] Benjamini, Y. & Hochberg, Y. Controlling the false discovery rate: A practical and powerful approach to multiple testing. *J. R. Stat. Soc. Ser. B Methodol.* **57**, 289–300 (1995).

[69] Hirose, S. et al. Efficiency of go/no-go task performance implemented in the left hemisphere. *J. Neurosci. Off. J. Soc. Neurosci.* **32**, 9059–9065 (2012). 10.1523/JNEUROSCI.0540-12.2012 PMC6622353 22745505

[70] Rubia, K. et al. Mapping motor inhibition: Conjunctive brain activations across different versions of Go/No-Go and stop tasks. *Neuroimage* **13**, 250–261 (2001). 11162266 10.1006/nimg.2000.0685

[71] Bunge, S. A., Dudukovic, N. M., Thomason, M. E., Vaidya, C. J. & Gabrieli, J. D. E. Immature frontal lobe contributions to cognitive control in children: Evidence from fMRI. *Neuron* **33**, 301–311 (2002). 11804576 10.1016/s0896-6273(01)00583-9 PMC4535916

[72] Johnstone, S. J., Pleffer, C. B., Barry, R. J., Clarke, A. R. & Smith, J. L. Development of inhibitory processing during the go/nogo task: A behavioral and event-related potential study of children and adults. *J. Psychophysiol.* **19**, 11–23 (2005).

[73] Juliano, J. M., Schweighofer, N. & Liew, S.-L. Increased cognitive load in immersive virtual reality during visuomotor adaptation is associated with decreased long-term retention and context transfer. *J. NeuroEngineering Rehabil.* **19**, 106 (2022). 10.1186/s12984-022-01084-6 PMC9532821 36199101

[74] Frederiksen, J. G. et al. Cognitive load and performance in immersive virtual reality versus conventional virtual reality simulation training of laparoscopic surgery: A randomized trial. *Surg. Endosc.* **34**, 1244–1252 (2020). 31172325 10.1007/s00464-019-06887-8

[75] Dong, D., Wong, L. K. F. & Luo, Z. Assess BA10 activity in slide-based and immersive virtual reality prospective memory task using functional near-infrared spectroscopy (fNIRS). *Appl. Neuropsychol. Adult* **26**, 465–471 (2019). 29547004 10.1080/23279095.2018.1443104

[76] Ge, R. et al. The effects of two game interaction modes on cortical activation in subjects of different ages: A functional near-infrared spectroscopy study. *IEEE Access* **9**, 11405–11415 (2021).

[77] Lorentz, L., Schüppen, A., Suchan, B. & Binkofski, F. Neural correlates of virtual reality-based attention training: An fMRI study. *Neuroimage* **284**, 120454 (2023). 37979896 10.1016/j.neuroimage.2023.120454

[78] Pinti, P. et al. The present and future use of functional near-infrared spectroscopy (fNIRS) for cognitive neuroscience. *Ann. N. Y. Acad. Sci.* **1464**, 5–29 (2020). 30085354 10.1111/nyas.13948 PMC6367070

[79] Pinti, P., Dina, L. M. & Smith, T. J. Ecological functional near-infrared spectroscopy in mobile children: Using short separation channels to correct for systemic contamination during naturalistic neuroimaging. *Neurophotonics* **11**, 045004 (2024). 39380715 10.1117/1.NPh.11.4.045004 PMC11460616

[80] Golding, J. F., Rafiq, A. & Keshavarz, B. Predicting individual susceptibility to visually induced motion sickness by questionnaire. *Front. Virtual Real.* **2**, 576871 (2021).

[81] Mühlberger, A. et al. The influence of methylphenidate on hyperactivity and attention deficits in children with ADHD: A virtual classroom test. *J. Atten. Disord.* **24**, 277–289 (2020). 27178061 10.1177/1087054716647480

[82] Clements, M. F. et al. Measuring trust with the Wayfinding Task: Implementing a novel task in immersive virtual reality and desktop setups across remote and in-person test environments. *PLoS ONE* **18**, e0294420 (2023). 38015928 10.1371/journal.pone.0294420 PMC10683989

[83] Yücel, M. A. et al. Best practices for fNIRS publications. *Neurophotonics* **8**, 012101 (2021). 33442557 10.1117/1.NPh.8.1.012101 PMC7793571

[84] Pinti, P., Dinu, L. & Smith, T. Ecological fNIRS in mobile children: Using short separation channels to correct for systemic contamination during naturalistic neuroimaging. 2024.06.02.596560 Preprint at 10.1101/2024.06.02.596560 (2024). 10.1117/1.NPh.11.4.045004 PMC11460616 39380715

[85] Zohdi, H., Egli, R., Guthruf, D., Scholkmann, F. & Wolf, U. Color-dependent changes in humans during a verbal fluency task under colored light exposure assessed by SPA-fNIRS. *Sci. Rep.* **11**, 9654 (2021). 33958616 10.1038/s41598-021-88059-0 PMC8102618

[86] Lloyd-Fox, S. et al. Coregistering functional near-infrared spectroscopy with underlying cortical areas in infants. *Neurophotonics* **1**, 025006 (2014). 25558463 10.1117/1.NPh.1.2.025006 PMC4280679

[87] Yeung, M. K. An optical window into brain function in children and adolescents: A systematic review of functional near-infrared spectroscopy studies. *Neuroimage* **227**, 117672 (2021). 33359349 10.1016/j.neuroimage.2020.117672

[88] Mehnert, J. et al. Developmental changes in brain activation and functional connectivity during response inhibition in the early childhood brain. *Brain Dev.* **35**, 894–904 (2013). 23265620 10.1016/j.braindev.2012.11.006

[89] Smith, E. et al. Prefrontal activation during executive tasks emerges over early childhood: Evidence from functional near infrared spectroscopy. *Dev. Neuropsychol.* **42**, 253–264 (2017). 28622028 10.1080/87565641.2017.1318391 PMC8074193

[90] Li, H., Subrahmanyam, K., Bai, X., Xie, X. & Liu, T. Viewing fantastical events versus touching fantastical events: Short-term effects on children’s inhibitory control. *CHILD Dev.* **89**, 48–57 (2018). 28478648 10.1111/cdev.12820

[91] Richards, J. E., Sanchez, C., Phillips-Meek, M. & Xie, W. A database of age-appropriate average MRI templates. *Neuroimage* **124**, 1254–1259 (2016). 25941089 10.1016/j.neuroimage.2015.04.055 PMC4630162

[92] Erel, Y., Jaffe-Dax, S., Yeshurun, Y. & Bermano, A. H. STORM-Net: Simple and timely optode registration method for functional near-infrared spectroscopy (fNIRS). 2020.12.29.424683 Preprint at 10.1101/2020.12.29.424683 (2021).

[93] Shattuck, D. W. et al. Construction of a 3D probabilistic atlas of human cortical structures. *Neuroimage* **39**, 1064–1080 (2008). 18037310 10.1016/j.neuroimage.2007.09.031 PMC2757616

[94] Patton, J. H., Stanford, M. S. & Barratt, E. S. Factor structure of the Barratt impulsiveness scale. *J. Clin. Psychol.* **51**, 768–774 (1995). 8778124 10.1002/1097-4679(199511)51:6<768::aid-jclp2270510607>3.0.co;2-1

[95] Kessler, R. C. et al. The World Health Organization adult ADHD self-report scale (ASRS): A short screening scale for use in the general population. *Psychol. Med.* **35**, 245–256 (2005). 15841682 10.1017/s0033291704002892

[96] Kessler, R. C. et al. Validity of the World Health Organization Adult ADHD Self-Report Scale (ASRS) Screener in a representative sample of health plan members. *Int. J. Methods Psychiatr. Res.* **16**, 52–65 (2007). 17623385 10.1002/mpr.208 PMC2044504

[97] Corbisiero, S., Hartmann-Schorro, R. M., Riecher-Rössler, A. & Stieglitz, R.-D. Screening for adult attention-deficit/hyperactivity disorder in a psychiatric outpatient population with specific focus on sex differences. *Front. Psychiatry* **8**, 115 (2017). 28713294 10.3389/fpsyt.2017.00115 PMC5491936

[98] Ramos-Quiroga, J. A. et al. Validation of the Spanish version of the attention deficit hyperactivity disorder adult screening scale (ASRS v. 1.1): A novel scoring strategy. *Rev. Neurol.* **48**, 449–452 (2009). 19396760

[99] American Psychological Association. *Diagnostic and Statistical Manual of Mental Disorders*. (2000).

[100] American Psychological Association. *Diagnostic and Statistical Manual of Mental Disorders*. (2013).

[101] Arnett, A. B. et al. The SWAN captures variance at both the negative and positive ends of the ADHD symptom dimension. *J. Atten. Disord.* **17**, 152–162 (2013). 22173148 10.1177/1087054711427399 PMC3330134

[102] Schulz-Zhecheva, Y. et al. ADHD traits in german school-aged children: Validation of the german strengths and weaknesses of ADHS Symptoms and normal behavior (SWAN-DE) scale. *J. Atten. Disord.* **23**, 553–562 (2019). 28043193 10.1177/1087054716676365

[103] Gioia, G. A., Isquith, P. K., Retzlaff, P. D. & Espy, K. A. Confirmatory factor analysis of the behavior rating inventory of executive function (BRIEF) in a clinical sample. *Child Neuropsychol. J. Norm. Abnorm. Dev. Child. Adolesc.* **8**, 249–257 (2002). 10.1076/chin.8.4.249.13513 12759822

[104] Kourtesis, P., Collina, S., Doumas, L. A. A. & MacPherson, S. E. Validation of the virtual reality neuroscience questionnaire: Maximum duration of immersive virtual reality sessions without the presence of pertinent adverse symptomatology. *Front. Hum. Neurosci.* **13**, 417 (2019). 31849627 10.3389/fnhum.2019.00417 PMC6901952

[105] Eldridge, S. M. et al. Defining feasibility and pilot studies in preparation for randomised controlled trials: Development of a conceptual framework. *PLoS ONE* **11**, e0150205 (2016). 26978655 10.1371/journal.pone.0150205 PMC4792418

[106] Sekhon, M., Cartwright, M. & Francis, J. J. Acceptability of healthcare interventions: An overview of reviews and development of a theoretical framework. *BMC Health Serv. Res.* **17**, 88 (2017). 28126032 10.1186/s12913-017-2031-8 PMC5267473

[107] Somrak, A., Pogačnik, M. & Guna, J. Suitability and comparison of questionnaires assessing virtual reality-induced symptoms and effects and user experience in virtual environments. *Sensors* **21**, 1185 (2021). 33567570 10.3390/s21041185 PMC7915458

[108] Kwak, S. K. & Kim, J. H. Statistical data preparation: Management of missing values and outliers. *Korean J. Anesthesiol.* **70**, 407–411 (2017). 28794835 10.4097/kjae.2017.70.4.407 PMC5548942

[109] Huppert, T. J., Diamond, S. G., Franceschini, M. A. & Boas, D. A. HomER: A review of time-series analysis methods for near-infrared spectroscopy of the brain. *Appl. Opt.* **48**, D280–D298 (2009). 19340120 10.1364/ao.48.00d280 PMC2761652

[110] Molavi, B. & Dumont, G. A. Wavelet-based motion artifact removal for functional near-infrared spectroscopy. *Physiol. Meas.* **33**, 259–270 (2012). 22273765 10.1088/0967-3334/33/2/259

[111] Scholkmann, F. & Wolf, M. General equation for the differential pathlength factor of the frontal human head depending on wavelength and age. *J. Biomed. Opt.* **18**, 105004 (2013). 24121731 10.1117/1.JBO.18.10.105004

[112] Zhao, H. et al. A wide field-of-view, modular, high-density diffuse optical tomography system for minimally constrained three-dimensional functional neuroimaging. *Biomed. Opt. Express* **11**, 4110 (2020). 32923032 10.1364/BOE.394914 PMC7449732

[113] Morimoto, S. & Minagawa, Y. Effects of hemodynamic differences on the assessment of inter-brain synchrony between adults and infants. *Front. Psychol.* **13**, 873796 (2022). 35719520 10.3389/fpsyg.2022.873796 PMC9205639

[114] Cusack, R. et al. Optimizing stimulation and analysis protocols for neonatal fMRI. *PLoS ONE* **10**, e0120202 (2015). 26266954 10.1371/journal.pone.0120202 PMC4534447

[115] Arichi, T. et al. Development of BOLD signal hemodynamic responses in the human brain. *Neuroimage* **63**, 663–673 (2012). 22776460 10.1016/j.neuroimage.2012.06.054 PMC3459097

[116] Brites, C., Salgado-Azoni, C. A., Ferreira, T. L., Lima, R. F. & Ciasca, S. M. Development and applications of the SWAN rating scale for assessment of attention deficit hyperactivity disorder: A literature review. *Braz. J. Med. Biol. Res.* **48**, 965–972 (2015). 26313140 10.1590/1414-431X20154528 PMC4671522

