# Supplementary material for: Measuring neurodevelopment of inhibitory control in children using naturalistic virtual reality
Source: Sci Rep. 2025 Jul 24;15:26944. doi: 10.1038/s41598-025-10974-3 (PMC12289916; doi:10.1038/s41598-025-10974-3)
Supplement: Supplementary file 1 — Supplementary Material 1 [file 41598_2025_10974_MOESM1_ESM.docx]

**Supplementary materials**

**Measuring neurodevelopment of inhibitory control in children using naturalistic virtual reality**

Larisa-Maria Dina*^1,2^, Paola Pinti^1, 3^, Tim J Smith^1,4^

^1^ Centre for Brain and Cognitive Development, Birkbeck, University of London WC1E 7JL, United Kingdom

^2^ Department of Psychology, King’s College London, London SE5 8AB, United Kingdom

^3^ Department of Medical Physics and Biomedical Engineering, University College London, United Kingdom

^4^ Creative Computing Institute, University of the Arts London, WC1V 7EY, United Kingdom

**Corresponding author:** Larisa-Maria Dina, Department of Psychology, King’s College London, London SE1 1UL, United Kingdom

E-mail: [larisa.dinu@kcl.ac.uk](mailto:larisa.dinu@kcl.ac.uk)

Spearman correlations between age, self-reports and task performance outcome measures in the adult sample.

| Variable 1 | Variable 2 | ρ [95% CI] | p | p (FDR) |
| --- | --- | --- | --- | --- |
| Age | BRIEF | -0.14 [-0.56, 0.31] | 0.503 | 0.865 |
| Age | VRISE | 0.04 [-0.37, 0.52] | 0.837 | 0.975 |
| Age | Emotional control | 0.01 [-0.44, 0.43] | 0.964 | 0.997 |
| Age | Inhibition | -0.14 [-0.53, 0.28] | 0.514 | 0.865 |
| Age | Plan/organise | -0.01 [-0.49, 0.43] | 0.947 | 0.997 |
| Age | Shift | -0.11 [-0.48, 0.31] | 0.611 | 0.865 |
| Age | Working memory | -0.09 [-0.55, 0.38] | 0.659 | 0.865 |
| Age | Error Rate Go (CB) | - | - | - |
| Age | Error Rate Mixed **(**VR**)** | -0.21 [-0.61, 0.22] | 0.329 | 0.734 |
| Age | Error Rate Go (VR) | 0.24 [-0.16, 0.58] | 0.261 | 0.656 |
| Age | Error Rate Mixed (CB) | 0 [-0.43, 0.46] | 0.997 | 0.997 |
| Age | RT Go **(**VR**)** | 0.13 [-0.31, 0.52] | 0.559 | 0.865 |
| Age | RT Go (CB) | 0.26 [-0.18, 0.69] | 0.222 | 0.597 |
| BRIEF | VRISE | -0.17 [-0.57, 0.28] | 0.428 | 0.857 |
| BRIEF | **Emotional control** | **0.81 [0.58, 0.94]** | **0.000** | **<.001***** |
| BRIEF | **Inhibition** | **0.85 [0.67, 0.93]** | **0.000** | **<.001***** |
| BRIEF | **Plan/organise** | **0.9 [0.73, 0.97]** | **0.000** | **<.001***** |
| BRIEF | **Shift** | **0.89 [0.73, 0.96]** | **0.000** | **<.001***** |
| BRIEF | **Working memory** | **0.88 [0.68, 0.97]** | **0.000** | **<.001***** |
| BRIEF | Error Rate Go (CB) | - | - | - |
| BRIEF | Error Rate Mixed **(**VR**)** | 0.08 [-0.33, 0.46] | 0.705 | 0.886 |
| BRIEF | Error Rate Go (VR) | 0.17 [-0.29, 0.56] | 0.416 | 0.854 |
| BRIEF | Error Rate Mixed (CB) | 0.23 [-0.19, 0.63] | 0.273 | 0.664 |
| BRIEF | RT Go **(**VR**)** | 0.28 [-0.2, 0.6] | 0.213 | 0.597 |
| BRIEF | RT Go (CB) | -0.17 [-0.55, 0.34] | 0.440 | 0.857 |
| VRISE | Emotional control | 0 [-0.42, 0.41] | 0.997 | 0.997 |
| VRISE | Inhibition | -0.25 [-0.6, 0.13] | 0.232 | 0.603 |
| VRISE | Plan/organise | 0 [-0.39, 0.41] | 0.996 | 0.997 |
| VRISE | Shift | -0.3 [-0.64, 0.12] | 0.155 | 0.525 |
| VRISE | Working memory | -0.2 [-0.61, 0.24] | 0.350 | 0.758 |
| VRISE | Error Rate Go (CB) | - | - | - |
| VRISE | Error Rate Mixed **(**VR**)** | 0.13 [-0.26, 0.52] | 0.550 | 0.865 |
| VRISE | Error Rate Go (VR) | -0.09 [-0.45, 0.33] | 0.677 | 0.865 |
| VRISE | Error Rate Mixed (CB) | -0.3 [-0.64, 0.05] | 0.153 | 0.525 |
| VRISE | RT Go **(**VR**)** | -0.09 [-0.48, 0.3] | 0.675 | 0.865 |
| VRISE | RT Go (CB) | 0.06 [-0.37, 0.46] | 0.770 | 0.915 |
| Emotional control | **Inhibition** | **0.64** [0.27, 0.88]** | **0.001** | **0.004**** |
| Emotional control | **Plan/organise** | **0.63** [0.22, 0.89]** | **0.001** | **0.005**** |
| Emotional control | **Shift** | **0.69** [0.39, 0.87]** | **0.000** | **0.001**** |
| Emotional control | **Working memory** | **0.69** [0.35, 0.91]** | **0.000** | **0.001**** |
| Emotional control | Error Rate Go (CB) | - | - | - |
| Emotional control | Error Rate Mixed **(**VR**)** | 0.03 [-0.37, 0.42] | 0.905 | 0.997 |
| Emotional control | Error Rate Go (VR) | -0.07 [-0.49, 0.36] | 0.732 | 0.898 |
| Emotional control | Error Rate Mixed (CB) | 0.26 [-0.11, 0.6] | 0.216 | 0.597 |
| Emotional control | RT Go **(**VR**)** | 0.11 [-0.34, 0.56] | 0.628 | 0.865 |
| Emotional control | RT Go (CB) | -0.12 [-0.51, 0.31] | 0.572 | 0.865 |
| Inhibition | **Plan/organise** | **0.78 [0.57, 0.87]** | **0.000** | **<.001***** |
| Inhibition | **Shift** | **0.77 [0.54, 0.9]** | **0.000** | **<.001***** |
| Inhibition | **Working memory** | **0.88 [0.69, 0.96]** | **0.000** | **<.001***** |
| Inhibition | Error Rate Go (CB) | - | - | - |
| Inhibition | Error Rate Mixed **(**VR**)** | 0.06 [-0.33, 0.42] | 0.774 | 0.915 |
| Inhibition | Error Rate Go (VR) | 0.01 [-0.35, 0.4] | 0.954 | 0.997 |
| Inhibition | Error Rate Mixed (CB) | 0.34 [-0.08, 0.69] | 0.109 | 0.426 |
| Inhibition | RT Go **(**VR**)** | 0.11 [-0.37, 0.56] | 0.628 | 0.865 |
| Inhibition | RT Go (CB) | -0.13 [-0.5, 0.31] | 0.541 | 0.865 |
| Plan/organise | Shift | 0.8 [0.56, 0.9] | 0.000 | <.001*** |
| Plan/organise | Working memory | 0.79 [0.52, 0.92] | 0.000 | <.001*** |
| Plan/organise | Error Rate Go (CB) | - | - | - |
| Plan/organise | Error Rate Mixed **(**VR**)** | 0.19 [-0.25, 0.54] | 0.372 | 0.785 |
| Plan/organise | Error Rate Go (VR) | 0.36 [-0.08, 0.7] | 0.087 | 0.358 |
| Plan/organise | Error Rate Mixed (CB) | 0.13 [-0.32, 0.53] | 0.543 | 0.865 |
| Plan/organise | RT Go **(**VR**)** | 0.43 [-0.02, 0.75] | 0.047 | 0.204 |
| Plan/organise | RT Go (CB) | -0.14 [-0.53, 0.33] | 0.513 | 0.865 |
| Shift | **Working memory** | **0.84 [0.65, 0.94]** | **0.000** | **<.001***** |
| Shift | Error Rate Go (CB) | - | - | - |
| Shift | Error Rate Mixed **(**VR**)** | -0.09 [-0.48, 0.29] | 0.662 | 0.865 |
| Shift | Error Rate Go (VR) | 0.27 [-0.13, 0.62] | 0.210 | 0.597 |
| Shift | Error Rate Mixed (CB) | 0.28 [-0.12, 0.63] | 0.190 | 0.592 |
| Shift | RT Go **(**VR**)** | 0.44 [0.02, 0.72] | 0.042 | 0.191 |
| Shift | RT Go (CB) | -0.11 [-0.49, 0.36] | 0.610 | 0.865 |
| Working memory | Error Rate Go (CB) | - | - | - |
| Working memory | Error Rate Mixed **(**VR**)** | -0.07 [-0.46, 0.36] | 0.737 | 0.898 |
| Working memory | Error Rate Go (VR) | 0.14 [-0.28, 0.5] | 0.506 | 0.865 |
| Working memory | Error Rate Mixed (CB) | 0.22 [-0.2, 0.62] | 0.293 | 0.678 |
| Working memory | RT Go **(**VR**)** | 0.33 [-0.11, 0.64] | 0.133 | 0.494 |
| Working memory | RT Go (CB) | -0.22 [-0.59, 0.25] | 0.295 | 0.678 |
| Error Rate Go (CB) | Error Rate Mixed **(**VR**)** | - | - | - |
| Error Rate Go (CB) | Error Rate Go (VR) | - | - | - |
| Error Rate Go (CB) | Error Rate Mixed (CB) | - | - | - |
| Error Rate Go (CB) | RT Go **(**VR**)** | - | - | - |
| Error Rate Go (CB) | RT Go **(**CB**)** | - | - | - |
| Error Rate Mixed (VR) | Error Rate Go (VR) | -0.15 [-0.54, 0.29] | 0.486 | 0.865 |
| Error Rate Mixed (VR) | Error Rate Mixed (CB) | -0.02 [-0.47, 0.41] | 0.941 | 0.997 |
| Error Rate Mixed (VR) | RT Go **(**VR**)** | -0.13 [-0.54, 0.34] | 0.572 | 0.865 |
| Error Rate Mixed (VR) | RT Go (CB) | -0.09 [-0.53, 0.34] | 0.676 | 0.865 |
| Error Rate Go (VR) | Error Rate Mixed (CB) | -0.11 [-0.45, 0.3] | 0.623 | 0.865 |
| Error Rate Go (VR) | **RT Go (VR)** | **0.58* [0.23, 0.79]** | **0.005** | **0.022*** |
| Error Rate Go (VR) | RT Go (CB) | 0.28 [-0.1, 0.61] | 0.178 | 0.577 |
| Error Rate Mixed (CB) | RT Go (CB) | 0.01 [-0.45, 0.48] | 0.953 | 0.997 |
| Error Rate Mixed (CB) | RT Go (CB) | 0.04 [-0.35, 0.4] | 0.865 | 0.989 |
| RT Go (VR) | RT Go (CB) | 0.04 [-0.36, 0.4] | 0.875 | 0.989 |

**Table S1***.* Spearman correlations between age, self-reports and task performance. All behavioural data was corrected for outliers as detailed in the Methods section of the main manuscript. The values in square brackets indicate the 95% confidence interval for each correlation. Both uncorrected and FDR-corrected p-values are presented for each correlation. * indicates *p* < .05. ** indicates *p* < .01.

**Convergent validity and discriminant validity**

Correlations between age, parent-reports and task performance outcome measures in the children sample.

| Variable 1 | Variable 2 | ρ [95% CI] | p | p (FDR) |
| --- | --- | --- | --- | --- |
| Age | SWAN | -0.02 [-0.34, 0.32] | 0.918 | 0.978 |
| Age | BRIEF | -0.16 [-0.54, 0.29] | 0.401 | 0.690 |
| Age | VRISE | -0.11 [-0.45, 0.24] | 0.539 | 0.803 |
| Age | Emotional control | -0.33 [-0.65, 0.02] | 0.071 | 0.225 |
| Age | Inhibition | -0.09 [-0.45, 0.29] | 0.638 | 0.837 |
| Age | Plan/organise | -0.17 [-0.56, 0.28] | 0.369 | 0.657 |
| Age | Shift | -0.17 [-0.56, 0.28] | 0.367 | 0.657 |
| Age | Working memory | -0.19 [-0.56, 0.23] | 0.317 | 0.584 |
| Age | **Error Rate Go (CB)** | **-0.46 [-0.64, -0.22]** | **0.005** | **0.031*** |
| Age | Error Rate Mixed (VR) | -0.13 [-0.45, 0.22] | 0.459 | 0.741 |
| Age | Error Rate Go (VR) | -0.35 [-0.66, 0] | 0.037 | 0.155 |
| Age | Error Rate Mixed (CB) | -0.05 [-0.4, 0.3] | 0.785 | 0.914 |
| Age | RT Go (VR) | 0.09 [-0.24, 0.41] | 0.604 | 0.830 |
| Age | RT Go (CB) | 0.08 [-0.29, 0.41] | 0.632 | 0.837 |
| SWAN | BRIEF | -0.22 [-0.59, 0.16] | 0.274 | 0.554 |
| SWAN | VRISE | -0.3 [-0.69, 0.13] | 0.124 | 0.327 |
| SWAN | Emotional control | 0.13 [-0.2, 0.45] | 0.543 | 0.803 |
| SWAN | Inhibition | -0.13 [-0.51, 0.29] | 0.525 | 0.803 |
| SWAN | Plan/organise | -0.16 [-0.47, 0.18] | 0.440 | 0.734 |
| SWAN | Shift | -0.24 [-0.63, 0.17] | 0.228 | 0.517 |
| SWAN | Working memory | -0.29 [-0.64, 0.08] | 0.154 | 0.376 |
| SWAN | Error Rate Go (CB) | 0.2 [-0.18, 0.55] | 0.285 | 0.554 |
| SWAN | Error Rate Mixed (VR) | -0.38 [-0.68, 0.03] | 0.040 | 0.157 |
| SWAN | Error Rate Go (VR) | 0.06 [-0.31, 0.41] | 0.750 | 0.914 |
| SWAN | Error Rate Mixed (CB) | -0.33 [-0.55, -0.05] | 0.070 | 0.225 |
| SWAN | RT Go (VR) | 0.41 [0.01, 0.72] | 0.029 | 0.132 |
| SWAN | RT Go (CB) | 0.11 [-0.24, 0.45] | 0.557 | 0.812 |
| BRIEF | VRISE | -0.15 [-0.49, 0.25] | 0.440 | 0.734 |
| BRIEF | **Emotional control** | **0.72 [0.43, 0.9]** | **0.000** | **0.000***** |
| BRIEF | **Inhibition** | **0.85 [0.66, 0.93]** | **0.000** | **0.000***** |
| BRIEF | **Plan/organise** | **0.82 [0.64, 0.91]** | **0.000** | **0.000***** |
| BRIEF | **Shift** | **0.68 [0.39, 0.84]** | **0.000** | **0.001***** |
| BRIEF | **Working memory** | **0.91 [0.79, 0.97]** | **0.000** | **0.000***** |
| BRIEF | **Error Rate Go (CB)** | 0.03 [-0.36, 0.41] | 0.864 | 0.958 |
| BRIEF | Error Rate Mixed (VR) | 0 [-0.35, 0.32] | 0.984 | 1.000 |
| BRIEF | Error Rate Go (VR) | -0.1 [-0.45, 0.27] | 0.586 | 0.828 |
| BRIEF | Error Rate Mixed (CB) | 0.31 [-0.1, 0.64] | 0.095 | 0.277 |
| BRIEF | RT Go (VR) | 0 [-0.37, 0.37] | 1.000 | 1.000 |
| BRIEF | **RT Go (CB)** | **-0.47 [-0.68, -0.18]** | **0.009** | **0.048*** |
| VRISE | Emotional control | -0.12 [-0.51, 0.27] | 0.513 | 0.803 |
| VRISE | Inhibition | -0.2 [-0.58, 0.2] | 0.278 | 0.554 |
| VRISE | Plan/organise | -0.03 [-0.41, 0.32] | 0.861 | 0.958 |
| VRISE | Shift | -0.2 [-0.53, 0.16] | 0.296 | 0.565 |
| VRISE | Working memory | -0.1 [-0.44, 0.27] | 0.591 | 0.828 |
| VRISE | Error Rate Go (CB) | -0.05 [-0.42, 0.33] | 0.792 | 0.914 |
| VRISE | Error Rate Mixed (VR) | 0.05 [-0.34, 0.44] | 0.769 | 0.914 |
| VRISE | Error Rate Go (VR) | -0.05 [-0.37, 0.29] | 0.792 | 0.914 |
| VRISE | Error Rate Mixed (CB) | -0.2 [-0.53, 0.17] | 0.268 | 0.554 |
| VRISE | RT Go (VR) | 0.05 [-0.35, 0.42] | 0.789 | 0.914 |
| VRISE | RT Go (CB) | 0.33 [-0.02, 0.61] | 0.064 | 0.215 |
| Emotional_control | **Inhibition** | **0.53 [0.17, 0.81]** | **0.002** | **0.019*** |
| Emotional_control | **Plan/organise** | **0.5 [0.09, 0.78]** | **0.005** | **0.031*** |
| Emotional_control | **Shift** | **0.61 [0.34, 0.81]** | **0.000** | **0.004**** |
| Emotional_control | **Working memory** | **0.58 [0.19, 0.83]** | **0.001** | **0.007**** |
| Emotional_control | Error Rate Go (CB) | 0.2 [-0.21, 0.62] | 0.284 | 0.554 |
| Emotional_control | Error Rate Mixed (VR) | -0.31 [-0.62, 0.06] | 0.090 | 0.271 |
| Emotional_control | Error Rate Go (VR) | 0.11 [-0.26, 0.45] | 0.578 | 0.828 |
| Emotional_control | Error Rate Mixed (CB) | 0.17 [-0.23, 0.56] | 0.383 | 0.671 |
| Emotional_control | RT Go (VR) | 0.26 [-0.09, 0.59] | 0.169 | 0.404 |
| Emotional_control | RT Go (CB) | -0.28 [-0.6, 0.09] | 0.132 | 0.339 |
| Inhibition | **Plan/organise** | **0.64 [0.33, 0.83]** | **0.000** | **0.001**** |
| Inhibition | **Shift** | **0.51 [0.12, 0.76]** | **0.004** | **0.029*** |
| Inhibition | **Working memory** | **0.69 [0.44, 0.85]** | **0.000** | **0.000***** |
| Inhibition | Error Rate Go (CB) | -0.03 [-0.41, 0.37] | 0.867 | 0.958 |
| Inhibition | Error Rate Mixed (VR) | 0.08 [-0.27, 0.44] | 0.665 | 0.862 |
| Inhibition | Error Rate Go (VR) | -0.05 [-0.4, 0.31] | 0.812 | 0.927 |
| Inhibition | Error Rate Mixed (CB) | 0.3 [-0.14, 0.62] | 0.112 | 0.317 |
| Inhibition | RT Go (VR) | -0.13 [-0.54, 0.28] | 0.517 | 0.803 |
| Inhibition | RT Go (CB) | -0.42 [-0.67, -0.07] | 0.021 | 0.106 |
| Plan/organise | Shift | 0.4 [0.07, 0.68] | 0.027 | 0.128 |
| Plan/organise | **Working memory** | **0.81 [0.62, 0.9]** | **0.000** | **0.000***** |
| Plan/organise | Error Rate Go (CB) | -0.02 [-0.4, 0.35] | 0.935 | 0.982 |
| Plan/organise | Error Rate Mixed (VR) | -0.02 [-0.4, 0.37] | 0.904 | 0.978 |
| Plan/organise | Error Rate Go (VR) | -0.28 [-0.59, 0.13] | 0.136 | 0.340 |
| Plan/organise | Error Rate Mixed (CB) | 0.29 [-0.15, 0.7] | 0.115 | 0.317 |
| Plan/organise | RT Go (VR) | -0.08 [-0.43, 0.29] | 0.698 | 0.894 |
| Plan/organise | RT Go (CB) | -0.39 [-0.65, -0.03] | 0.035 | 0.155 |
| Shift | **Working memory** | **0.52 [0.16, 0.77]** | **0.003** | **0.026*** |
| Shift | Error Rate Go (CB) | 0.21 [-0.12, 0.55] | 0.266 | 0.554 |
| Shift | Error Rate Mixed (VR) | 0.12 [-0.2, 0.42] | 0.539 | 0.803 |
| Shift | Error Rate Go (VR) | 0.14 [-0.22, 0.45] | 0.451 | 0.739 |
| Shift | Error Rate Mixed (CB) | 0.22 [-0.16, 0.55] | 0.247 | 0.541 |
| Shift | RT Go (VR) | -0.06 [-0.42, 0.31] | 0.754 | 0.914 |
| Shift | RT Go (CB) | -0.29 [-0.62, 0.12] | 0.124 | 0.327 |
| Working memory | Error Rate Go (CB) | 0.02 [-0.39, 0.42] | 0.922 | 0.978 |
| Working memory | Error Rate Mixed (VR) | 0.05 [-0.3, 0.39] | 0.780 | 0.914 |
| Working memory | Error Rate Go (VR) | -0.23 [-0.56, 0.14] | 0.232 | 0.517 |
| Working memory | Error Rate Mixed (CB) | 0.37 [-0.01, 0.66] | 0.042 | 0.157 |
| Working memory | RT Go (VR) | -0.03 [-0.38, 0.34] | 0.876 | 0.958 |
| Working memory | **RT Go (CB)** | **-0.5 [-0.71, -0.18]** | **0.005** | **0.031*** |
| Error Rate Go (CB) | Error Rate Mixed (VR) | 0.2 [-0.18, 0.52] | 0.231 | 0.517 |
| Error Rate Go (CB) | Error Rate Go (VR) | 0.41 [0.07, 0.68] | 0.014 | 0.073 |
| Error Rate Go (CB) | Error Rate Mixed (CB) | 0.09 [-0.27, 0.45] | 0.612 | 0.830 |
| Error Rate Go (CB) | RT Go (VR) | -0.32 [-0.6, 0.05] | 0.062 | 0.215 |
| Error Rate Go (CB) | RT Go (CB) | 0 [-0.36, 0.36] | 0.994 | 1.000 |
| Error Rate Mixed (VR) | Error Rate Go (VR) | 0.06 [-0.33, 0.4] | 0.709 | 0.897 |
| Error Rate Mixed (VR) | Error Rate Mixed (CB) | 0.3 [-0.07, 0.58] | 0.078 | 0.241 |
| Error Rate Mixed (VR) | **RT Go (VR)** | **-0.66 [-0.82, -0.39]** | **0.000** | **0.000***** |
| Error Rate Mixed (VR) | RT Go (CB) | -0.33 [-0.58, 0] | 0.050 | 0.183 |
| Error Rate Go (VR) | Error Rate Mixed (CB) | 0.09 [-0.26, 0.45] | 0.617 | 0.830 |
| Error Rate Go (VR) | RT Go (VR) | -0.01 [-0.37, 0.35] | 0.974 | 1.000 |
| Error Rate Go (VR) | RT Go (CB) | -0.01 [-0.35, 0.31] | 0.966 | 1.000 |
| Error Rate Mixed (CB) | RT Go (VR) | -0.35 [-0.61, 0] | 0.042 | 0.157 |
| Error Rate Mixed (CB) | **RT Go (CB)** | **-0.55 [-0.75, -0.25]** | **0.001** | **0.005**** |
| RT Go (VR) | RT Go (CB) | 0.18 [-0.19, 0.5] | 0.301 | 0.565 |

**Table S2***.* Spearman correlations between age, parent-reports and task performance. All behavioural data was corrected for outliers as detailed in the Methods section of the main manuscript. The values in square brackets indicate the 95% confidence interval for each correlation. Both uncorrected and FDR-corrected p-values are presented for each correlation. * indicates *p* < .05. ** indicates *p* < .01. *** indicates *p* < .001.

**Exploratory analyses**

Impulsivity in children (SWAN)

The sample of toddlers and pre-schoolers was split into low and high impulsivity based on a median split on the SWAN total score, as no established cut-off score exists (median = 74). We had SWAN data from 30 participants. Based on the median split approach, 60% (N = 18) of the sample with valid SWAN data (N = 30) were classified as highly impulsive (M = 79, SD = 4.95) and 40% as low in impulsivity (M = 68.83, SD = 5.72). Children higher in impulsivity had longer reaction times in the CAVE task (p_FDR corrected_ = .048). There were no other significant differences in terms of task performance outcomes between low and high impulsive children.

|  | **Group** | **Mean (SD)** | **Z** | **p** | **p (FDR)** |
| --- | --- | --- | --- | --- | --- |
| Error rate Go blocks (CAVE) | Low impulsivity | .08 (.07) | -.26 | .798 | .865 |
|  | High impulsivity | .08 (.10) |  |  |  |
| Error rate Mixed blocks (CAVE) | Low impulsivity | .50 (.17) | -2.06 | .040 | .120 |
|  | High impulsivity | .35 (.22) |  |  |  |
| **Reaction time Go blocks (CAVE)** | **Low impulsivity** | **.73 (.16)** | **-2.66** | **.008** | **.048*** |
|  | **High impulsivity** | **.92 (.16)** |  |  |  |
| Error rate Go blocks (computer task) | Low impulsivity | .10 (.12) | -.17 | .865 | .865 |
|  | High impulsivity | .10 (.13) |  |  |  |
| Error rate Mixed blocks (computer task) | Low impulsivity | .19 (.13) | -1.54 | .125 | .250 |
|  | High impulsivity | .12 (.12) |  |  |  |
| Reaction time Go blocks  (computer task) | Low impulsivity | .83 (.15) | -.59 | .553 | .830 |
|  | High impulsivity | .90 (.23) |  |  |  |

**Table S3**. Mann-Whitney U tests comparing outcome variables from the CAVE and standardised computer task between low and high impulsive toddlers and pre-schoolers based on a median split on total SWAN scores.

Impulsivity in adults (BIS)

The sample of adults was split into low and high impulsivity using a cut-off of 72 for the Barrett Impulsiveness Scale. It is important to note that only 12.5% of the sample were considered highly impulsive based on this cut-off score (M = 75, SD = 1.41). There were no differences between different impulsivity profiles and task performance outcomes in the computer or novel CAVE tasks.

|  | **Group** | **Mean (SD)** | **Z** | **p** | **p (FDR)** |
| --- | --- | --- | --- | --- | --- |
| Error rate Go blocks (CAVE) | Low impulsivity | .008 (.012) | -.26 | .795 | 1 |
|  | High impulsivity | .011 (.019) |  |  |  |
| Error rate Mixed blocks (CAVE) | Low impulsivity | .037 (.041) | -.23 | .817 | 1 |
|  | High impulsivity | .022 (.019) |  |  |  |
| Reaction time Go blocks (CAVE) | Low impulsivity | 1.09 (.285) | -34 | .738 | 1 |
|  | High impulsivity | 1.09 (.411) |  |  |  |
| Error rate Go blocks (computer task) | Low impulsivity | .000 (.000) | .00 | 1.00 | 1 |
|  | High impulsivity | .000 (.000) |  |  |  |
| Error rate Mixed blocks (computer task) | Low impulsivity | .028 (.034) | -.05 | .962 | 1 |
|  | High impulsivity | .022 (.019) |  |  |  |
| Reaction time Go blocks  (computer task) | Low impulsivity | .749 (.308) | -.92 | .401 | 1 |
|  | High impulsivity | .522 (.136) |  |  |  |

**Table S4**. Mann-Whitney U tests comparing outcome variables from the CAVE and standardised computer task between low and high impulsive adults based on a median split on total BIS scores.

**Anatomical locations for fNIRS channels**

|  | LPBA40 label | | Probability | | N_CAVE_ | | N_CB_ | |
| --- | --- | --- | --- | --- | --- | --- | --- | --- |
| Channel | **Children** | **Adults** | **Children** | **Adults** | **Children** | **Adults** | **Children** | **Adults** |
| Ch 1 | R middle frontal gyrus | R middle frontal gyrus | 0.84 | 1 | 28 | 20 | 24 | 20 |
| Ch 2 | R middle frontal gyrus | R middle frontal gyrus | 0.88 | 0.24 | 29 | 18 | 28 | 17 |
|  |  | R inferior frontal gyrus |  | 0.76 |  |  |  |  |
| Ch 3 | R middle frontal gyrus | R middle frontal gyrus | 0.99 | 1 | 25 | 19 | 24 | 20 |
| Ch 4 | R middle frontal gyrus | R middle frontal gyrus | 0.86 | 0.94 | 20 | 20 | 20 | 16 |
| Ch 5 | R superior frontal gyrus | R middle frontal gyrus | 0.65 | 0.46 | 19 | 20 | 13 | 20 |
|  | R middle frontal gyrus | R inferior frontal gyrus | 0.35 | 0.54 |  |  |  |  |
| Ch 6 | R middle frontal gyrus | R middle frontal gyrus | 0.96 | 0.88 | 30 | 20 | 27 | 18 |
| Ch 7 | R middle frontal gyrus | R inferior frontal gyrus | 0.93 | 0.98 | 26 | 17 | 25 | 19 |
| Ch 8 | R inferior frontal gyrus | R inferior frontal gyrus | 0.76 | 0.57 | 27 | 18 | 30 | 20 |
|  |  | R precentral frontal gyrus |  | 0.43 |  |  |  |  |
| Ch 9 | R precentral gyrus | R superior frontal gyrus | 0.75 | 0.31 | 25 | 8 | 26 | 4 |
|  |  | R middle frontal gyrus |  | 0.69 |  |  |  |  |
| Ch 10 | R superior frontal gyrus | R middle frontal gyrus | 0.75 | 1 | 17 | 14 | 15 | 8 |
|  | R middle frontal gyrus |  | 0.24 |  |  |  |  |  |
| Ch 11 | R precentral gyrus | R middle frontal gyrus | 0.70 | 0.86 | 27 | 13 | 23 | 10 |
| Ch 12 | R postcentral gyrus | R superior frontal gyrus | 0.57 | 0.47 | 25 | 8 | 22 | 5 |
|  | R precentral gyrus | R middle frontal gyrus | 0.32 | 0.53 |  |  |  |  |
| Ch 13 | R precentral gyrus | R superior frontal gyrus | 0.78 | 0.55 | 13 | 7 | 14 | 6 |
|  |  | R middle frontal gyrus |  | 0.26 |  |  |  |  |
| Ch 14 | L precentral gyrus | L superior frontal gyrus | 0.53 | 0.69 | 17 | 4 | 17 | 4 |
|  | L postcentral gyrus | L middle frontal gyrus | 0.43 | 0.29 |  |  |  |  |
| Ch 15 | L precentral gyrus | L superior frontal gyrus | 0.57 | 0.69 | 22 | 6 | 21 | 4 |
|  | L middle frontal gyrus | L middle frontal gyrus | 0.37 | 0.31 |  |  |  |  |
| Ch 16 | L middle frontal gyrus | L middle frontal gyrus | 0.76 | 1 | 13 | 10 | 11 | 11 |
| Ch 17 | L precentral gyrus | L middle frontal gyrus | 0.70 | 1 | 15 | 11 | 15 | 12 |
| Ch 18 | L middle frontal gyrus | L superior frontal gyrus | 0.90 | 0.46 | 28 | 9 | 27 | 7 |
|  |  | L middle frontal gyrus |  | 0.54 |  |  |  |  |
| Ch 19 | L middle frontal gyrus | L middle frontal gyrus | 0.85 | 1 | 19 | 17 | 15 | 16 |
| Ch 20 | L middle frontal gyrus | L middle frontal gyrus | 0.95 | 0.87 | 28 | 16 | 26 | 12 |
| Ch 21 | L middle frontal gyrus | L middle frontal gyrus | 0.94 | 1 | 23 | 19 | 18 | 16 |
| Ch 22 | L inferior frontal gyrus | L middle frontal gyrus | 0.48 | 1 | 20 | 19 | 26 | 19 |
|  | L precentral gyrus |  | 0.38 |  |  |  |  |  |
| Ch 23 | L inferior frontal gyrus | L inferior frontal gyrus | 0.70 | 0.88 | 22 | 16 | 30 | 17 |
|  | L middle frontal gyrus |  | 0.29 |  |  |  |  |  |
| Ch 24 | L middle frontal gyrus | L inferior frontal gyrus | 0.83 | 0.96 | 28 | 20 | 28 | 20 |
| Ch 25 | L middle frontal gyrus | L middle frontal gyrus | 0.97 | 0.71 | 25 | 19 | 22 | 20 |
|  |  | L inferior frontal gyrus |  | 0.29 |  |  |  |  |
| Ch 26 | L middle frontal gyrus | L middle frontal gyrus | 0.95 | 1 | 22 | 16 | 20 | 19 |

**Table S5.** Anatomical locations of the fNIRS channels. The anatomical areas (LPBA40 atlas) and the corresponding atlas-based probabilities for each channel are included. Only probabilities greater than 20% are listed. Number of participants contributing to each channel for the CAVE (N_CAVE_ and computer-based (N_CB_) tasks because of cap placement and data quality are also listed.

**Block-by-block behavioural performance in children**


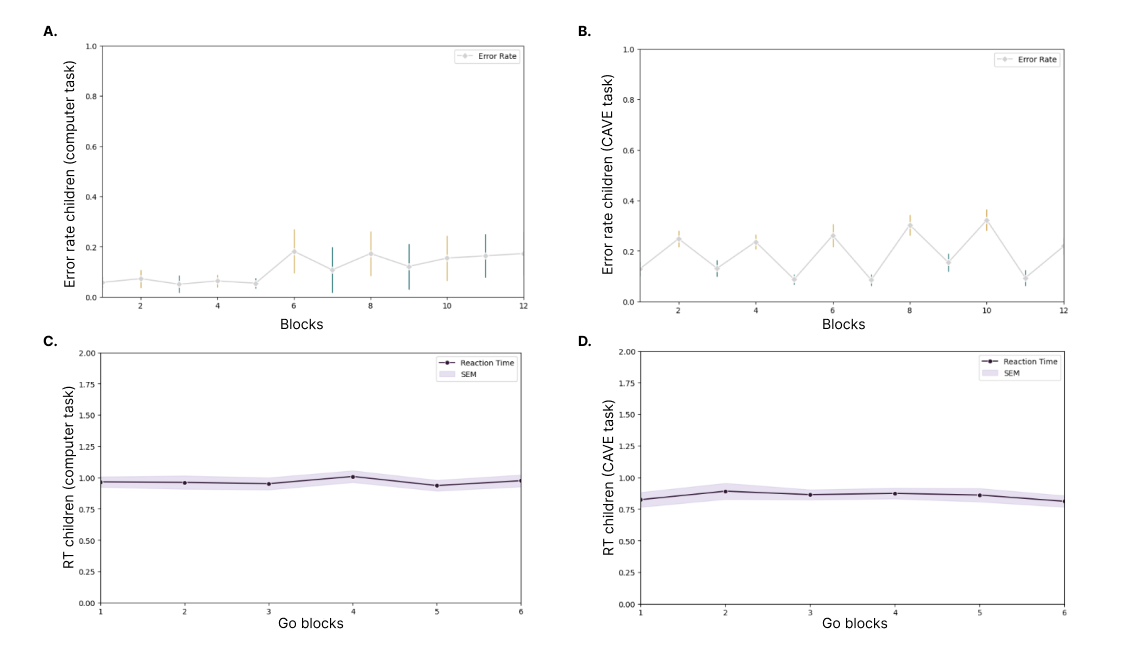


**Figure S1.** Block-by-block changes in (A) error rates in the computer task, (B) error rates in the CAVE task, (C) reaction time in the computer task, and (D) reaction time in the CAVE task in children.

|  | **Estimate** | **Std. Error** | **df** | **t** | **p** |
| --- | --- | --- | --- | --- | --- |
| (Intercept) | 0.06 | 0.07 | 33.01 | 0.83 | 0.41 |
| Block2 | 0.01 | 0.07 | 110.00 | 0.21 | 0.83 |
| Block3 | - 0.01 | 0.07 | 110.00 | 0.10 | 0.92 |
| Block4 | 0.01 | 0.07 | 110.00 | 0.08 | 0.94 |
| Block5 | 0.00 | 0.07 | 110.00 | -0.05 | 0.96 |
| Block6 | 0.12 | 0.07 | 110.00 | 1.75 | 0.08 |
| Block7 | 0.05 | 0.07 | 110.00 | 0.70 | 0.49 |
| Block8 | 0.11 | 0.07 | 110.00 | 1.62 | 0.11 |
| Block9 | 0.06 | 0.07 | 110.00 | 0.89 | 0.37 |
| Block10 | 0.10 | 0.07 | 110.00 | 1.37 | 0.17 |
| Block11 | 0.11 | 0.07 | 110.00 | 1.49 | 0.14 |
| Block12 | 0.11 | 0.07 | 110.00 | 1.62 | 0.11 |

**Table S6**. Error rate by block (1-12) in the computer task in children.

|  | **Estimate** | **Std. Error** | **df** | **t** | **p** |
| --- | --- | --- | --- | --- | --- |
| (Intercept) | 0.13 | 0.03 | 201.47 | 3.91 | 0.00 |
| **Block2** | **0.12** | **0.04** | **337.52** | **2.98** | **0.00***** |
| Block3 | 0.00 | 0.04 | 337.52 | 0.05 | 0.96 |
| **Block4** | **0.11** | **0.04** | **337.52** | **2.68** | **0.01**** |
| Block5 | -0.04 | 0.04 | 337.52 | -0.91 | 0.37 |
| **Block6** | **0.14** | **0.04** | **337.52** | **3.39** | **0.00***** |
| Block7 | -0.04 | 0.04 | 337.52 | -0.96 | 0.34 |
| **Block8** | **0.18** | **0.04** | **337.52** | **4.42** | **0.00***** |
| Block9 | 0.03 | 0.04 | 337.52 | 0.76 | 0.45 |
| **Block10** | **0.20** | **0.04** | **337.52** | **4.89** | **0.00***** |
| Block11 | -0.03 | 0.04 | 337.52 | -0.74 | 0.46 |
| **Block12** | **0.10** | **0.04** | **337.52** | **2.36** | **0.02**** |

***, p<.001; **, p<.01.

**Table S7**. Error rate by block (1-12) in the CAVE task in children.

|  | **Estimate** | **Std. Error** | **df** | **t** | **p** |
| --- | --- | --- | --- | --- | --- |
| (intercept) | 0.82 | 0.05 | 165.06 | 16.17 | 0.00 |
| Block2 | 0.07 | 0.06 | 180.00 | 1.08 | 0.28 |
| Block3 | 0.04 | 0.06 | 180.00 | 0.64 | 0.52 |
| Block4 | 0.05 | 0.06 | 180.00 | 0.80 | 0.43 |
| Block5 | 0.04 | 0.06 | 180.00 | 0.59 | 0.56 |
| Block6 | -0.01 | 0.06 | 180.00 | -0.21 | 0.84 |

**Table S8**. Reaction time by block (1-12) in the CAVE task in children.

|  | **Estimate** | **Std. Error** | **df** | **t-** | **p** |
| --- | --- | --- | --- | --- | --- |
| (intercept) | 0.97 | 0.05 | 80.05 | 20.82 | 0.00 |
| Block2 | 0.00 | 0.04 | 184.75 | -0.10 | 0.92 |
| Block3 | -0.01 | 0.04 | 184.75 | -0.35 | 0.73 |
| Block4 | 0.03 | 0.04 | 184.75 | 0.83 | 0.41 |
| Block5 | -0.04 | 0.04 | 184.75 | -0.93 | 0.35 |
| Block6 | 0.01 | 0.04 | 184.75 | 0.20 | 0.84 |

**Table S9**. Reaction time by block (1-12) in the computer task in children.

**Correlations between behavioural performance and brain activity in children**

Spearman correlations were run for error rates and reaction time, and the channels which we found to be significantly activated in the fNIRS analysis before multiple comparison corrections. Specifically, for the children group in the computer task we ran correlations between behavioural performance (error rates and RTs) and channels 11 and 23 for HbO and HbR; in the VR task, we ran correlations between behavioural performance (error rates and RTs) and channels 2, 6, 7, 9, 14, 15, 23. For the adult group, in the computer task we ran correlations between behavioural performance (error rates and RTs) and channels 9, 13, and 18; in the VR task, we ran correlations between behavioural performance (error rates and RTs) and channels 16, 19, 24.

| Task | Block | Variable | Chromophore | Channel | r | p | p (FDR) |
| --- | --- | --- | --- | --- | --- | --- | --- |
| CB | Go | Error Rate | HbO | 11 | -0.26 | <.001 | <.001*** |
| CB | Go | Error Rate | HbO | 23 | -0.19 | <.001 | <.001*** |
| CB | Go | Error Rate | HbR | 11 | -0.17 | 0.001 | 0.002** |
| CB | Go | Error Rate | HbR | 23 | -0.29 | <.001 | <.001*** |
| CB | Go | RT | HbO | 11 | -0.33 | <.001 | <.001*** |
| CB | Go | RT | HbO | 23 | 0.08 | 0.103 | 0.138 |
| CB | Go | RT | HbR | 11 | -0.42 | <.001 | <.001*** |
| CB | Go | RT | HbR | 23 | -0.13 | 0.010 | 0.017* |
| CB | Mixed | Error Rate | HbO | 11 | 0.24 | <.001 | <.001*** |
| CB | Mixed | Error Rate | HbO | 23 | 0.25 | <.001 | <.001*** |
| CB | Mixed | Error Rate | HbR | 11 | -0.15 | 0.005 | 0.007** |
| CB | Mixed | Error Rate | HbR | 23 | 0.20 | <.001 | <.001*** |
| CB | Mixed | RT | HbO | 11 | 0.55 | <.001 | <.001*** |
| CB | Mixed | RT | HbO | 23 | 0.29 | <.001 | <.001*** |
| CB | Mixed | RT | HbR | 11 | 0.01 | 0.791 | 0.863 |
| CB | Mixed | RT | HbR | 23 | 0.04 | 0.407 | 0.473 |
| VR | Go | Error Rate | HbO | 2 | 0.03 | 0.517 | 0.600 |
| VR | Go | Error Rate | HbO | 6 | 0.048 | 0.297 | 0.356 |
| VR | Go | Error Rate | HbO | 7 | 0.13 | 0.007 | 0.014* |
| VR | Go | Error Rate | HbO | 9 | 0.14 | 0.005 | 0.010* |
| VR | Go | Error Rate | HbO | 14 | -0.17 | 0.013 | 0.021* |
| VR | Go | Error Rate | HbO | 15 | -0.24 | <.001 | <.001*** |
| VR | Go | Error Rate | HbO | 23 | 0.12 | 0.024 | 0.038* |
| VR | Go | Error Rate | HbR | 2 | 0.21 | <.001 | <.001*** |
| VR | Go | Error Rate | HbR | 6 | 0.07 | 0.103 | 0.138 |
| VR | Go | Error Rate | HbR | 7 | 0.13 | 0.007 | 0.014* |
| VR | Go | Error Rate | HbR | 9 | -0.13 | 0.008 | 0.014* |
| VR | Go | Error Rate | HbR | 14 | 0.41 | <.001 | <.001*** |
| VR | Go | Error Rate | HbR | 15 | 0.10 | 0.035 | 0.052 |
| VR | Go | Error Rate | HbR | 23 | -0.11 | 0.038 | 0.055 |
| VR | Go | RT | HbO | 2 | -0.28 | <.001 | <.001*** |
| VR | Go | RT | HbO | 6 | 0.02 | 0.619 | 0.692 |
| VR | Go | RT | HbO | 7 | 0.02 | 0.634 | 0.692 |
| VR | Go | RT | HbO | 9 | -0.30 | <.001 | <.001*** |
| VR | Go | RT | HbO | 14 | -0.68 | <.001 | <.001*** |
| VR | Go | RT | HbO | 15 | -0.05 | 0.276 | 0.343 |
| VR | Go | RT | HbO | 23 | -0.02 | 0.759 | 0.781 |
| VR | Go | RT | HbR | 2 | 0.42 | <.001 | <.001*** |
| VR | Go | RT | HbR | 6 | 0.40 | <.001 | <.001*** |
| VR | Go | RT | HbR | 7 | 0.24 | <.001 | <.001*** |
| VR | Go | RT | HbR | 9 | -0.02 | 0.721 | 0.764 |
| VR | Go | RT | HbR | 14 | 0.01 | 0.875 | 0.875 |
| VR | Go | RT | HbR | 15 | 0.06 | 0.210 | 0.270 |
| VR | Go | RT | HbR | 23 | 0.25 | <.001 | <.001*** |
| VR | Mixed | Error Rate | HbO | 2 | -0.24 | <.001 | <.001*** |
| VR | Mixed | Error Rate | HbO | 6 | -0.23 | <.001 | <.001*** |
| VR | Mixed | Error Rate | HbO | 7 | 0.01 | 0.888 | 0.903 |
| VR | Mixed | Error Rate | HbO | 9 | 0.03 | 0.590 | 0.664 |
| VR | Mixed | Error Rate | HbO | 14 | 0.01 | 0.903 | 0.903 |
| VR | Mixed | Error Rate | HbO | 15 | -0.10 | 0.039 | 0.052 |
| VR | Mixed | Error Rate | HbO | 23 | -0.14 | 0.008 | 0.011* |
| VR | Mixed | Error Rate | HbR | 2 | 0.21 | <.001 | <.001*** |
| VR | Mixed | Error Rate | HbR | 6 | 0.36 | <.001 | <.001*** |
| VR | Mixed | Error Rate | HbR | 7 | 0.24 | <.001 | <.001*** |
| VR | Mixed | Error Rate | HbR | 9 | 0.15 | 0.003 | 0.005** |
| VR | Mixed | Error Rate | HbR | 14 | 0.27 | <.001 | <.001*** |
| VR | Mixed | Error Rate | HbR | 15 | -0.31 | <.001 | <.001*** |
| VR | Mixed | Error Rate | HbR | 23 | 0.16 | 0.002 | 0.003** |
| VR | Mixed | RT | HbO | 2 | -0.17 | <.001 | <.001*** |
| VR | Mixed | RT | HbO | 6 | -0.21 | <.001 | <.001*** |
| VR | Mixed | RT | HbO | 7 | -0.27 | <.001 | <.001*** |
| VR | Mixed | RT | HbO | 9 | -0.33 | <.001 | <.001*** |
| VR | Mixed | RT | HbO | 14 | 0.57 | <.001 | <.001*** |
| VR | Mixed | RT | HbO | 15 | -0.34 | <.001 | <.001*** |
| VR | Mixed | RT | HbO | 23 | -0.34 | <.001 | <.001*** |
| VR | Mixed | RT | HbR | 2 | -0.23 | <.001 | <.001*** |
| VR | Mixed | RT | HbR | 6 | -0.21 | <.001 | <.001*** |
| VR | Mixed | RT | HbR | 7 | -0.09 | 0.072 | 0.090 |
| VR | Mixed | RT | HbR | 9 | -0.05 | 0.316 | 0.380 |
| VR | Mixed | RT | HbR | 14 | -0.13 | 0.069 | 0.089 |
| VR | Mixed | RT | HbR | 15 | -0.01 | 0.899 | 0.903 |
| VR | Mixed | RT | HbR | 23 | 0.19 | <.001 | <.001*** |

**Table S10**. Spearman correlations between behavioural performance and significant fNIRS channels in children. All behavioural data was corrected for outliers as detailed in the Methods section of the main manuscript. Both uncorrected and FDR-corrected p-values are presented for each correlation. * indicates p < .05. ** indicates p < .01. *** indicates p < .001.

| Task | Block | Variable | Chromophore | Channel | r | p | p (FDR) |
| --- | --- | --- | --- | --- | --- | --- | --- |
| CB | Go | Error Rate | HbO | 9 | nan | NA | nan |
| CB | Go | Error Rate | HbO | 13 | nan | NA | nan |
| CB | Go | Error Rate | HbO | 18 | nan | NA | nan |
| CB | Go | Error Rate | HbR | 9 | nan | NA | nan |
| CB | Go | Error Rate | HbR | 13 | nan | NA | nan |
| CB | Go | Error Rate | HbR | 18 | nan | NA | nan |
| CB | Go | RT | HbO | 9 | 0.5 | 0.002 | 0.003** |
| CB | Go | RT | HbO | 13 | 0.13 | 0.133 | 0.163 |
| CB | Go | RT | HbO | 18 | 0.31 | <.001 | 0.001** |
| CB | Go | RT | HbR | 9 | -1.0 | <.001 | <.001*** |
| CB | Go | RT | HbR | 13 | 0.34 | <.001 | <.001*** |
| CB | Go | RT | HbR | 18 | -0.21 | 0.016 | 0.026* |
| CB | Mixed | Error Rate | HbO | 9 | nan | NA | nan |
| CB | Mixed | Error Rate | HbO | 13 | nan | NA | nan |
| CB | Mixed | Error Rate | HbO | 18 | nan | NA | nan |
| CB | Mixed | Error Rate | HbR | 9 | nan | NA | nan |
| CB | Mixed | Error Rate | HbR | 13 | nan | NA | nan |
| CB | Mixed | Error Rate | HbR | 18 | nan | NA | nan |
| CB | Mixed | RT | HbO | 9 | -0.5 | 0.002 | 0.003** |
| CB | Mixed | RT | HbO | 13 | 0.20 | 0.019 | 0.030* |
| CB | Mixed | RT | HbO | 18 | -0.47 | <.001 | <.001*** |
| CB | Mixed | RT | HbR | 9 | -1.0 | <.001 | <.001*** |
| CB | Mixed | RT | HbR | 13 | 0.30 | <.001 | 0.001** |
| CB | Mixed | RT | HbR | 18 | 0.78 | <.001 | <.001*** |
| VR | Go | Error Rate | HbO | 16 | nan | NA | nan |
| VR | Go | Error Rate | HbO | 19 | 0.16 | 0.050 | 0.073 |
| VR | Go | Error Rate | HbO | 24 | 0.46 | <.001 | <.001*** |
| VR | Go | Error Rate | HbR | 16 | nan | NA | nan |
| VR | Go | Error Rate | HbR | 19 | -0.03 | 0.745 | 0.745 |
| VR | Go | Error Rate | HbR | 24 | -0.09 | 0.288 | 0.329 |
| VR | Go | RT | HbO | 16 | 0.66 | <.001 | <.001*** |
| VR | Go | RT | HbO | 19 | 0.14 | 0.087 | 0.116 |
| VR | Go | RT | HbO | 24 | 0.73 | <.001 | <.001*** |
| VR | Go | RT | HbR | 16 | 0.09 | 0.474 | 0.506 |
| VR | Go | RT | HbR | 19 | -0.34 | <.001 | <.001*** |
| VR | Go | RT | HbR | 24 | -0.31 | <.001 | <.001*** |
| VR | Mixed | Error Rate | HbO | 16 | nan | NA | nan |
| VR | Mixed | Error Rate | HbO | 19 | -0.16 | 0.050 | 0.057 |
| VR | Mixed | Error Rate | HbO | 24 | 0.17 | 0.033 | 0.040* |
| VR | Mixed | Error Rate | HbR | 16 | nan | NA | nan |
| VR | Mixed | Error Rate | HbR | 19 | -0.52 | <.001 | <.001*** |
| VR | Mixed | Error Rate | HbR | 24 | -0.17 | 0.033 | 0.040* |
| VR | Mixed | RT | HbO | 16 | 0.26 | 0.029 | 0.040* |
| VR | Mixed | RT | HbO | 19 | 0.25 | 0.002 | 0.003** |
| VR | Mixed | RT | HbO | 24 | 0.53 | <.001 | <.001*** |
| VR | Mixed | RT | HbR | 16 | -0.03 | 0.812 | 0.812 |
| VR | Mixed | RT | HbR | 19 | -0.29 | <.001 | 0.001** |
| VR | Mixed | RT | HbR | 24 | 0.07 | 0.413 | 0.441 |

**Table S11**. Spearman correlations between behavioural performance and significant fNIRS channels in adults. All behavioural data was corrected for outliers as detailed in the Methods section of the main manuscript. Both uncorrected and FDR-corrected p-values are presented for each correlation. * indicates p < .05. ** indicates p < .01. *** indicates p < .001.

**Correlations between self- and parent-reports and brain activity**

Spearman correlations were run for the BRIEF and SWAN (total scores) and the channels which we found to be significantly activated in the fNIRS analyses of the computer and CAVE tasks before multiple comparison corrections. Specifically, for the children group in the computer task we ran correlations between the questionnaires and channels 11 and 23 for HbO and HbR; in the VR task, we ran correlations between the questionnaires and channels 2, 6, 7, 9, 14, 15, 23. For the adult group, in the computer task we ran correlations between the questionnaires and channels 9, 13, and 18; in the VR task, we ran correlations between the questionnaires and channels 16, 19, 24.

| Task | Block | Variable | Chromophore | Channel | r | p | p (FDR) |
| --- | --- | --- | --- | --- | --- | --- | --- |
| CB | Go | BRIEF | HbO | 11 | 0.23 | <.001 | <.001*** |
| CB | Go | BRIEF | HbO | 23 | -0.41 | <.001 | <.001*** |
| CB | Go | BRIEF | HbR | 11 | -0.05 | 0.406 | 0.443 |
| CB | Go | BRIEF | HbR | 23 | -0.10 | 0.068 | 0.09 |
| CB | Go | SWAN | HbO | 11 | -0.32 | <.001 | <.001*** |
| CB | Go | SWAN | HbO | 23 | 0.15 | 0.009 | 0.014 |
| CB | Go | SWAN | HbR | 11 | 0.07 | 0.193 | 0.232 |
| CB | Go | SWAN | HbR | 23 | 0.01 | 0.891 | 0.891 |
| CB | Mixed | BRIEF | HbO | 11 | -0.39 | <.001 | <.001*** |
| CB | Mixed | BRIEF | HbO | 23 | 0.01 | 0.800 | 0.823 |
| CB | Mixed | BRIEF | HbR | 11 | 0.14 | 0.012 | 0.022 |
| CB | Mixed | BRIEF | HbR | 23 | -0.03 | 0.623 | 0.661 |
| CB | Mixed | SWAN | HbO | 11 | 0.37 | <.001 | <.001*** |
| CB | Mixed | SWAN | HbO | 23 | 0.46 | <.001 | <.001*** |
| CB | Mixed | SWAN | HbR | 11 | 0.29 | <.001 | <.001*** |
| CB | Mixed | SWAN | HbR | 23 | 0.29 | <.001 | <.001*** |
| VR | Go | BRIEF | HbO | 2 | 0.33 | <.001 | <.001*** |
| VR | Go | BRIEF | HbO | 6 | 0.31 | <.001 | <.001*** |
| VR | Go | BRIEF | HbO | 7 | 0.21 | <.001 | <.001*** |
| VR | Go | BRIEF | HbO | 9 | 0.24 | <.001 | <.001*** |
| VR | Go | BRIEF | HbO | 14 | -0.85 | <.001 | <.001*** |
| VR | Go | BRIEF | HbO | 15 | 0.11 | 0.031 | 0.041 |
| VR | Go | BRIEF | HbO | 23 | 0.08 | 0.166 | 0.206 |
| VR | Go | BRIEF | HbR | 2 | -0.52 | <.001 | <.001*** |
| VR | Go | BRIEF | HbR | 6 | -0.17 | <.001 | 0.001** |
| VR | Go | BRIEF | HbR | 7 | 0.31 | <.001 | <.001*** |
| VR | Go | BRIEF | HbR | 9 | -0.18 | <.001 | <.001*** |
| VR | Go | BRIEF | HbR | 14 | -0.17 | 0.022 | 0.031 |
| VR | Go | BRIEF | HbR | 15 | -0.11 | 0.029 | 0.040* |
| VR | Go | BRIEF | HbR | 23 | -0.04 | 0.506 | 0.536 |
| VR | Go | SWAN | HbO | 2 | -0.20 | <.001 | <.001*** |
| VR | Go | SWAN | HbO | 6 | -0.05 | 0.318 | 0.358 |
| VR | Go | SWAN | HbO | 7 | -0.05 | 0.317 | 0.358 |
| VR | Go | SWAN | HbO | 9 | -0.15 | 0.011 | 0.017 |
| VR | Go | SWAN | HbO | 14 | 0.46 | <.001 | <.001*** |
| VR | Go | SWAN | HbO | 15 | -0.24 | <.001 | <.001*** |
| VR | Go | SWAN | HbO | 23 | 0.20 | 0.001 | 0.002 |
| VR | Go | SWAN | HbR | 2 | 0.21 | <.001 | <.001*** |
| VR | Go | SWAN | HbR | 6 | 0.19 | <.001 | <.001*** |
| VR | Go | SWAN | HbR | 7 | -0.01 | 0.886 | 0.891 |
| VR | Go | SWAN | HbR | 9 | 0.26 | <.001 | <.001*** |
| VR | Go | SWAN | HbR | 14 | 0.31 | <.001 | <.001*** |
| VR | Go | SWAN | HbR | 15 | 0.18 | <.001 | 0.001 |
| VR | Go | SWAN | HbR | 23 | 0.38 | <.001 | <.001*** |
| VR | Mixed | BRIEF | HbO | 2 | -0.16 | 0.001 | 0.003 |
| VR | Mixed | BRIEF | HbO | 6 | 0.19 | <.001 | <.001*** |
| VR | Mixed | BRIEF | HbO | 7 | 0.14 | 0.006 | 0.013* |
| VR | Mixed | BRIEF | HbO | 9 | 0.15 | 0.004 | 0.010* |
| VR | Mixed | BRIEF | HbO | 14 | -0.07 | 0.336 | 0.418 |
| VR | Mixed | BRIEF | HbO | 15 | 0.03 | 0.549 | 0.638 |
| VR | Mixed | BRIEF | HbO | 23 | -0.19 | <.001 | 0.002** |
| VR | Mixed | BRIEF | HbR | 2 | -0.05 | 0.316 | 0.406 |
| VR | Mixed | BRIEF | HbR | 6 | 0.12 | 0.012 | 0.022* |
| VR | Mixed | BRIEF | HbR | 7 | -0.13 | 0.011 | 0.021* |
| VR | Mixed | BRIEF | HbR | 9 | 0.04 | 0.410 | 0.492 |
| VR | Mixed | BRIEF | HbR | 14 | -0.33 | <.001 | <.001*** |
| VR | Mixed | BRIEF | HbR | 15 | -0.06 | 0.196 | 0.272 |
| VR | Mixed | BRIEF | HbR | 23 | -0.56 | <.001 | <.001*** |
| VR | Mixed | SWAN | HbO | 2 | 0.06 | 0.216 | 0.287 |
| VR | Mixed | SWAN | HbO | 6 | -0.13 | 0.014 | 0.024* |
| VR | Mixed | SWAN | HbO | 7 | -0.13 | 0.021 | 0.033* |
| VR | Mixed | SWAN | HbO | 9 | -0.03 | 0.624 | 0.661 |
| VR | Mixed | SWAN | HbO | 14 | -0.39 | <.001 | <.001*** |
| VR | Mixed | SWAN | HbO | 15 | -0.10 | 0.049 | 0.071 |
| VR | Mixed | SWAN | HbO | 23 | -0.27 | <.001 | <.001*** |
| VR | Mixed | SWAN | HbR | 2 | -0.35 | <.001 | <.001*** |
| VR | Mixed | SWAN | HbR | 6 | -0.38 | <.001 | <.001*** |
| VR | Mixed | SWAN | HbR | 7 | -0.13 | 0.018 | 0.029* |
| VR | Mixed | SWAN | HbR | 9 | -0.03 | 0.592 | 0.661 |
| VR | Mixed | SWAN | HbR | 14 | 0.0 | 1.000 | 1.0 |
| VR | Mixed | SWAN | HbR | 15 | 0.26 | <.001 | <.001*** |
| VR | Mixed | SWAN | HbR | 23 | 0.13 | 0.033 | 0.050 |

**Table S12.** Spearman correlations between parent-reports (SWAN and BRIEF total scores) and significant fNIRS channels in children. All behavioural data was corrected for outliers as detailed in the Methods section of the main manuscript. Both uncorrected and FDR-corrected p-values are presented for each correlation. * indicates p < .05. ** indicates p < .01. *** indicates p < .001.

| Task | Block | Variable | Chromophore | Channel | r | p | p (FDR) |
| --- | --- | --- | --- | --- | --- | --- | --- |
| CB | Go | ASRS (Part A) | HbO | 9 | -0.5 | 0.002 | 0.005** |
| CB | Go | ASRS (Part A) | HbO | 13 | 0 | 0.426 | 0.512 |
| CB | Go | ASRS (Part A) | HbO | 18 | -0.22 | 0.012 | 0.021* |
| CB | Go | ASRS (Part A) | HbR | 9 | -0.5 | 0.002 | 0.005** |
| CB | Go | ASRS (Part A) | HbR | 13 | -0.17 | 0.043 | 0.061 |
| CB | Go | ASRS (Part A) | HbR | 18 | 0.23 | 0.008 | 0.017* |
| CB | Go | BRIEF | HbO | 9 | 0.0 | 1.000 | 1.0 |
| CB | Go | BRIEF | HbO | 13 | 0.06 | 0.453 | 0.518 |
| CB | Go | BRIEF | HbO | 18 | -0.25 | 0.004 | 0.011* |
| CB | Go | BRIEF | HbR | 9 | -0.87 | <.001 | <.001*** |
| CB | Go | BRIEF | HbR | 13 | -0.16 | 0.054 | 0.072 |
| CB | Go | BRIEF | HbR | 18 | 0.19 | 0.028 | 0.044* |
| CB | Mixed | ASRS (Part A) | HbO | 9 | 0.5 | 0.002 | 0.004** |
| CB | Mixed | ASRS (Part A) | HbO | 13 | -0.24 | 0.004 | 0.006** |
| CB | Mixed | ASRS (Part A) | HbO | 18 | 0.47 | <.001 | <.001*** |
| CB | Mixed | ASRS (Part A) | HbR | 9 | -0.5 | 0.002 | 0.004** |
| CB | Mixed | ASRS (Part A) | HbR | 13 | -0.58 | <.001 | <.001*** |
| CB | Mixed | ASRS (Part A) | HbR | 18 | -0.28 | 0.001 | 0.003** |
| CB | Mixed | BRIEF | HbO | 9 | 0.0 | 1.000 | 1.0 |
| CB | Mixed | BRIEF | HbO | 13 | -0.16 | 0.054 | 0.076 |
| CB | Mixed | BRIEF | HbO | 18 | 0.40 | <.001 | <.001*** |
| CB | Mixed | BRIEF | HbR | 9 | -0.87 | <.001 | <.001*** |
| CB | Mixed | BRIEF | HbR | 13 | -0.56 | <.001 | <.001*** |
| CB | Mixed | BRIEF | HbR | 18 | -0.11 | 0.212 | 0.268 |
| VR | Go | ASRS (Part A) | HbO | 16 | 0.20 | 0.092 | 0.116 |
| VR | Go | ASRS (Part A) | HbO | 19 | 0.25 | 0.002 | 0.005** |
| VR | Go | ASRS (Part A) | HbO | 24 | 0.50 | <.001 | <.001*** |
| VR | Go | ASRS (Part A) | HbR | 16 | -0.94 | <.001 | <.001*** |
| VR | Go | ASRS (Part A) | HbR | 19 | 0.02 | 0.811 | 0.885 |
| VR | Go | ASRS (Part A) | HbR | 24 | -0.33 | <.001 | <.001*** |
| VR | Go | BRIEF | HbO | 16 | 0.26 | 0.029 | 0.044* |
| VR | Go | BRIEF | HbO | 19 | 0.18 | 0.026 | 0.044* |
| VR | Go | BRIEF | HbO | 24 | 0.41 | <.001 | <.001*** |
| VR | Go | BRIEF | HbR | 16 | -1.0 | <.001 | <.001*** |
| VR | Go | BRIEF | HbR | 19 | -0.01 | 0.892 | 0.930 |
| VR | Go | BRIEF | HbR | 24 | -0.23 | 0.005 | 0.011* |
| VR | Mixed | ASRS (Part A) | HbO | 16 | 0.6 | <.001 | <.001*** |
| VR | Mixed | ASRS (Part A) | HbO | 19 | -0.06 | 0.452 | 0.517 |
| VR | Mixed | ASRS (Part A) | HbO | 24 | 0.33 | <.001 | <.001*** |
| VR | Mixed | ASRS (Part A) | HbR | 16 | -0.14 | 0.231 | 0.278 |
| VR | Mixed | ASRS (Part A) | HbR | 19 | -0.44 | <.001 | <.001*** |
| VR | Mixed | ASRS (Part A) | HbR | 24 | 0.17 | 0.033 | 0.050 |
| VR | Mixed | BRIEF | HbO | 16 | 0.77 | <.001 | <.001*** |
| VR | Mixed | BRIEF | HbO | 19 | -0.06 | 0.495 | 0.517 |
| VR | Mixed | BRIEF | HbO | 24 | 0.14 | 0.087 | 0.116 |
| VR | Mixed | BRIEF | HbR | 16 | -0.09 | 0.474 | 0.517 |
| VR | Mixed | BRIEF | HbR | 19 | -0.44 | <.001 | <.001*** |
| VR | Mixed | BRIEF | HbR | 24 | 0.24 | 0.004 | 0.006** |

**Table S13**. Spearman correlations between parent-reports (Part A of the ASRS and BRIEF total scores) and significant fNIRS channels in adults. All behavioural data was corrected for outliers as detailed in the Methods section of the main manuscript. Both uncorrected and FDR-corrected p-values are presented for each correlation. * indicates p < .05. ** indicates p < .01. *** indicates p < .001.

| **Task** | **Block** | **Trial type** | **Group** | **Mean (SD)** | **Range** |
| --- | --- | --- | --- | --- | --- |
| Computer | Go-Only | Go | Children | 56.31 (8.16) | 30-60 |
|  | Mixed | Go | Children | 27.92 (4.53) | 10-30 |
|  | Mixed | No-Go | Children | 27.92 (4.53) | 10-30 |
| CAVE | Go-Only | Go | Children | 57.63 (5.61) | 39-60 |
|  | Mixed | Go | Children | 27.86 (4.25) | 15-30 |
|  | Mixed | No-Go | Children | 27.86 (4.25) | 15-30 |
| Computer | Go-Only | Go | Adults | 60 (0) | 60 |
|  | Mixed | Go | Adults | 30 (0) | 30 |
|  | Mixed | No-Go | Adults | 30 (0) | 30 |
| CAVE | Go-Only | Go | Adults | 60 (0) | 60 |
|  | Mixed | Go | Adults | 30 (0) | 30 |
|  | Mixed | No-Go | Adults | 30 (0) | 30 |

**Table S14**. Number of Go and No-Go trials included in the analysis for each block type, each task and each developmental group.
